# LCM-seq reveals unique transcriptional adaptation mechanisms of resistant neurons and identifies protective pathways in spinal muscular atrophy

**DOI:** 10.1101/gr.265017.120

**Published:** 2020-08

**Authors:** Susanne Nichterwitz, Jik Nijssen, Helena Storvall, Christoph Schweingruber, Laura Helen Comley, Ilary Allodi, Mirjam van der Lee, Qiaolin Deng, Rickard Sandberg, Eva Hedlund

**Affiliations:** 1 Department of Neuroscience, Karolinska Institutet, 171 77 Stockholm, Sweden;; 2 Department of Cell and Molecular Biology, Karolinska Institutet, 171 77 Stockholm, Sweden;; 3 Ludwig Institute for Cancer Research, Karolinska Institutet, 171 77 Stockholm, Sweden

## Abstract

Somatic motor neurons are selectively vulnerable in spinal muscular atrophy (SMA), which is caused by a deficiency of the ubiquitously expressed survival of motor neuron protein. However, some motor neuron groups, including oculomotor and trochlear (ocular), which innervate eye muscles, are for unknown reasons spared. To reveal mechanisms of vulnerability and resistance in SMA, we investigate the transcriptional dynamics in discrete neuronal populations using laser capture microdissection coupled with RNA sequencing (LCM-seq). Using gene correlation network analysis, we reveal a TRP53-mediated stress response that is intrinsic to all somatic motor neurons independent of their vulnerability, but absent in relatively resistant red nucleus and visceral motor neurons. However, the temporal and spatial expression analysis across neuron types shows that the majority of SMA-induced modulations are cell type–specific. Using Gene Ontology and protein network analyses, we show that ocular motor neurons present unique disease-adaptation mechanisms that could explain their resilience. Specifically, ocular motor neurons up-regulate (1) *Syt1*, *Syt5*, and *Cplx2*, which modulate neurotransmitter release; (2) the neuronal survival factors *Gdf15, Chl1*, and *Lif*; (3) *Aldh4,* that protects cells from oxidative stress; and (4) the caspase inhibitor *Pak4.* Finally, we show that GDF15 can rescue vulnerable human spinal motor neurons from degeneration. This confirms that adaptation mechanisms identified in resilient neurons can be used to reduce susceptibility of vulnerable neurons. In conclusion, this in-depth longitudinal transcriptomics analysis in SMA reveals novel cell type–specific changes that, alone and combined, present compelling targets, including *Gdf15*, for future gene therapy studies aimed toward preserving vulnerable motor neurons.

Spinal muscular atrophy (SMA) is an autosomal recessive disease characterized by the progressive degeneration of somatic motor neurons in spinal cord and lower brainstem. SMA is caused by the loss of functional survival motor neuron (SMN) protein caused by loss of or mutations in the telomeric gene *SMN1*. SMA displays a wide clinical spectrum and is classified based on age of onset and severity of the disease. An increased copy number of the centromeric *SMN2* gene is the main predictor of disease severity (Lorson et al. 1999; Vitali et al. 1999; Wirth 2000; Feldkötter et al. 2002). *SMN1* and *SMN2* differ by five nucleotides only and would encode identical proteins. However, a C to T nucleotide transition in exon 7 of the *SMN2* gene disrupts an exonic splicing enhancer and leads to alternative splicing, in which a majority of *SMN2* transcripts lack exon 7 (*SMNΔ7*) (Lorson et al. 1999; Monani et al. 1999). Although SMNΔ7 appears to be a functional SMN protein, it is highly unstable and quickly degraded (Cho and Dreyfuss 2010). Recently, the first drug treatment for SMA, based on increasing full-length SMN through *SMN2* exon 7 inclusion, was approved. This presents a very promising therapeutic strategy for the broad treatment of SMA, with positive outcomes in several clinical studies (Finkel et al. 2017; Pechmann et al. 2018). However, the timing of the initiation of treatment appears crucial for the outcome, and patients will very likely benefit from additional treatments that aim to preserve or improve motor function.

The best characterized function of SMN is its role in the assembly of small nuclear ribonucleoproteins (snRNPs), which are major components of the pre-mRNA splicing machinery (Fischer et al. 1997). SMN can be found in nuclear gems and in the cytoplasm but in neurons it is also located in axons and growth cones (Pagliardini et al. 2000; Jablonka et al. 2001; Giavazzi et al. 2006). A more widespread role for SMN in RNP assembly is now accepted owing to the disruption of axonal mRNA localization and translation in an SMN-deficient context (Donlin-Asp et al. 2016). There is also strong evidence that SMN can prevent DNA damage and apoptosis (Vyas et al. 2002; Sareen et al. 2012; Zhao et al. 2016). As of now, it remains unclear which of these functions, when disrupted, lead to SMA.

SMN is ubiquitously expressed and its complete depletion leads to early embryonic lethality (Schrank et al. 1997). In SMA, somatic motor neurons are for unknown reasons selectively vulnerable to the lower level of SMN protein. However, different somatic motor neuron groups show varying degrees of susceptibility to degeneration. Spinal motor neurons are the primarily affected cell type in disease. Facial motor neurons and hypoglossal motor neurons that innervate the tongue are to some extent affected in severe cases of the human disease (Rudnik-Schöneborn et al. 2009; Petit et al. 2011; Harding et al. 2015). Neuromuscular junctions (NMJs), the specialized synapses between motor neurons and muscle, of facial motor neurons present pathology in mouse models of SMA (Murray et al. 2008) while hypoglossal NMJs remain unaffected (Comley et al. 2016). Ocular motor neurons, which innervate extraocular muscles and thus control movement of the eyes, appear consistently resistant in SMA. This is evidenced by the use of ocular tracking devices as a communication tool for patients (Kubota et al. 2000) and that NMJs are preserved in extraocular muscles of end-stage SMA mice (Comley et al. 2016).

Genes active within specific neuronal types define their unique identities and functions in health as well as their susceptibility to specific neurodegenerative diseases. Data from SMA animal models and SMA patient motor neurons derived from induced pluripotent stem cells (iPSCs) indicate that factors intrinsic to motor neurons are important for degeneration (Park et al. 2010; Corti et al. 2012; Van Hoecke et al. 2012). Thus, investigating cell intrinsic pathways that are differentially activated within resistant and vulnerable motor neurons could reveal mechanisms of selective neuronal degeneration and lead to therapies preventing progressive motor neuron loss (Hedlund et al. 2010; Kaplan et al. 2014; Comley et al. 2015; Murray et al. 2015; Allodi et al. 2016; Nijssen et al. 2017; Allodi et al. 2019).

Previous transcriptome studies in SMA have compared vulnerable patient-derived motor neurons (Ng et al. 2015) or whole spinal cords isolated from SMA mice (Zhang et al. 2008; Bäumer et al. 2009; Murray et al. 2010; Staropoli et al. 2015) with healthy controls. These studies have improved our understanding of motor neuron disease mechanisms but could not explain how the loss of a ubiquitously expressed protein can induce degeneration in a select cell type. Zhang et al. (2013) included unaffected cells from the white matter in their analysis and a more recent study by Murray et al. (2015) investigated transcriptional changes in differentially affected motor neuron pools. However, these studies were restricted to a single presymptomatic stage, limiting the scope of the findings. To unravel temporal mechanisms of neuronal resilience and susceptibility, we have conducted a comprehensive longitudinal analysis of resistant and vulnerable neuron groups from a presymptomatic stage to early and late symptomatic stages. We used laser capture microdissection coupled with RNA sequencing (LCM-seq) (Nichterwitz et al. 2016, 2018) to profile discrete neuronal populations in SMA mice and littermate controls over time.

## Results

### Transcriptional profiling of neurons with differential susceptibility to degeneration reveals cell type–specific gene expression

**Figure 1.**
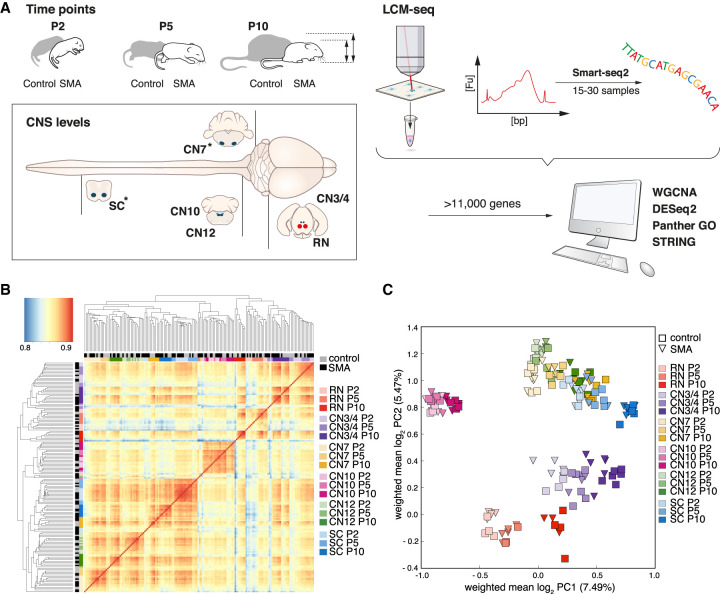
LCM-seq strategy to reveal cell intrinsic mechanisms of motor neuron vulnerability and resistance in SMA. (*A*) We used “delta 7” mice (*Smn*^−/−^/*SMN2*^+/+^/*SMNΔ7*^+/+^) as a model for severe SMA and littermates homozygous for murine *Smn* as controls (*Smn*^+/+^/*SMN2*^+/+^/*SMNΔ7*^+/+^). (*B*) Pairwise Spearman's correlation on log_10_-transformed data of all samples per cell type, genotype, and age. (*C*) Principal component analysis on the whole gene expression data set. (P) Postnatal day; (CNS) central nervous system; (LCM-seq) laser capture microdissection coupled with RNA sequencing; (SC) spinal cord; (CN) cranial nerve; (CN12) hypoglossal nucleus; (CN10) dorsal motor nucleus of the vagus nerve; (CN7) facial nucleus; (CN3/4) oculomotor and trochlear nuclei; (RN) red nucleus; asterisks in *A* indicate vulnerable cell types in this mouse model; (WGCNA) weighted gene correlation network analysis; (GO) Gene Ontology.

To investigate the transcriptional dynamics of neuronal populations with differential vulnerability in SMA, we used the widely studied “delta7” mouse model (*Smn*^−/−^/*SMN2*^+/+^/*SMNΔ7*^+/+^). Because we were interested in longitudinal changes in gene expression, we analyzed several disease stages. We included a presymptomatic stage (P2) and an early symptomatic stage (P5) when motor neuron loss in this model is restricted to discrete regions of the spinal cord (toward rostral and medial levels) (Mentis et al. 2011). We also included a symptomatic stage (P10) when the *Smn*-deficient mice have clear motor dysfunction and show a more widespread loss of spinal motor neurons (Fig. 1A; Le et al. 2005; Mentis et al. 2011). We applied laser capture microdissection (LCM) to isolate neurons from different regions of the brainstem and spinal cord (Fig. 1A; Supplemental Fig. S1) coupled with poly(A)-enriched RNA sequencing (LCM-seq) (Nichterwitz et al. 2016, 2018). We collected somatic motor neurons from the oculomotor and trochlear nuclei [cranial nuclei 3 and 4 (CN3/4)] (Supplemental Fig. S1D–F) and the hypoglossal nucleus [cranial nucleus 12 (CN12)] (Supplemental Fig. S1M–O) that are resistant to degeneration in this SMA model. We also isolated relatively vulnerable somatic motor neurons from the facial nucleus [cranial nucleus 7 (CN7)] (Supplemental Fig. S1G–I) and along the lumbar spinal cord (SC) (Supplemental Figs. S1P–R, S2A,B). To ensure the inclusion of vulnerable neurons within motor neuron populations with mixed vulnerabilities over time, for example, CN7 neurons innervating the rostral versus the caudal band of the levator auris longus muscle (Murray et al. 2008) or neurons in the medial and lateral motor columns in the spinal cord (Mentis et al. 2011), we collected cells across the entire motor neuron nuclei.

Furthermore, we collected visceral motor neurons from the dorsal motor nucleus of the vagus nerve (vagus motor neurons) [cranial nucleus 10 (CN10)] (Supplemental Fig. S1J–L) to deduct events occurring within all cholinergic motor neurons versus those specific for somatic motor neurons. Visceral motor neurons are generally considered more resilient to SMA disease processes than somatic motor neurons and were thus also included as a relatively resistant neuronal population. We also isolated red nucleus neurons (RN) (Supplemental Fig. S1A–C), which are noncholinergic neurons involved in motor coordination that are resilient to degeneration in SMA, to elucidate disease-induced transcriptional regulation selective to cholinergic neurons versus more broad regulation across neuronal populations. We thus acquired an extensive library of six neuronal populations at three different time points throughout disease progression in health and SMA.

After conducting LCM-seq, we performed a quality control, using only samples in which we achieved a gene detection level of greater than 11,000 expressed genes (Supplemental Fig. S3A), which left a total of 168 samples for further analysis (Supplemental Table S1). To evaluate the purity of the LCM-seq samples, we analyzed the level of neuronal and glial markers and compared these to a published RNA sequencing data set of neurons, astrocytes, oligodendrocytes, and microglia (NCBI Gene Expression Omnibus [GEO] accession number GSE52564) (Zhang et al. 2014). The neuronal markers neurofilament heavy chain (*Nefh*) and peripherin (*Prph*) were highly expressed in all LCM-seq samples. The motor neuron markers choline acetyl transferase (*Chat*) and Islet-1/2 were readily detected in all motor neuron groups, whereas *Hb9* (*Mnx1*) expression was largely restricted to SC and CN12 motor neurons. Glial markers, *Gfap*, *Mfge8*, *Sox10*, *Pdgfrb*, *Enpp6*, and *Mog* were detectable in all neuron samples, but at much lower levels than in glial populations, whereas the macrophage/microglia markers *Itgam* (*CD11b*) and *Ccl3* were absent from our neuronal samples (Supplemental Fig. S3C). This cross-comparison showed that the LCM-seq samples were highly enriched in neuronal transcripts and only included minor contaminations of glial transcripts. We analyzed the sustained homeobox transcription factor (Hox) gene profiles of collected neurons (Hedlund et al. 2010; Nichterwitz et al. 2016), which confirmed their anterior-posterior positions along the body axis. RN and CN3/4 neurons, which are located in the midbrain were, as expected, devoid of Hox gene expression (Supplemental Fig. S3D). Together, these data confirmed the identity, purity, and high quality of the LCM-seq neuronal samples.

To investigate reproducibility among biological replicate samples and to determine correlations between different neuron types, we conducted pairwise Spearman's correlation. This analysis showed a high correlation between samples originating from the same neuron type and revealed a close relationship between SC, CN7, and CN12 motor neurons (Fig. 1B). After unsupervised hierarchical clustering, midbrain neurons (RN and CN3/4) were distinct from other brainstem and SC motor neurons, and visceral CN10 motor neurons clustered separately within this group (Fig. 1B). Principal component analysis (PCA) on the entire gene expression data set revealed clustering of cell types with a strong influence of their developmental origin (Fig. 1C). Consequently, CN3/4 motor neurons clustered closely to RN neurons consistent with their specification in the ventral midbrain (Deng et al. 2011). CN7, CN12, and SC motor neurons formed a dense cluster, whereas visceral CN10 motor neurons clustered distinct from all the other cell populations. Altogether, we could show that biological replicates show high reproducibility and that neuronal groups form distinct clusters indicative of a high sensitivity.

In-depth gene expression analysis across neuron types confirmed known marker gene expression and identified several novel cell type–specific transcripts. We confirmed their mRNA localization in the adult central nervous system using the Allen Mouse Brain Atlas (available at https://portal.brain-map.org). We could show that *Cxcl13* was restricted to RN neurons (Supplemental Fig. S4A). The transcription factor *Lhx4* was preferentially expressed in CN3/4 motor neurons (Supplemental Fig. S4B), but *Shox2* was present in CN7 motor neurons (Supplemental Fig. S4C). The peptide hormone *Nppb* distinguished visceral CN10 motor neurons from the other cell types (Supplemental Fig. S4D), whereas the proteoglycan *Dcn* was a marker for CN12 motor neurons (Supplemental Fig. S4E). We could thus validate the cell type–specific expression from the RNA-seq analysis with available in situ data and identify unique cell type–specific markers.

Spinal motor neurons display inefficient *SMN2* exon 7 inclusion compared to other cells in the spinal cord, thus rendering these cells low in full-length SMN (Ruggiu et al. 2012). To investigate if *SMN2* splicing differences could account for the differential susceptibility among the neuron types investigated here, we first specifically examined the expression levels of full-length *SMN2* mRNA by qPCR (Supplemental Fig. S5A). In many of the samples we could not detect any exon 7 inclusion, and where we did, the signal was close to the detection threshold (Supplemental Fig. S5B). We also analyzed the RNA-seq data for exon 7 inclusion. By pooling aligned reads from several samples within each neuron group, we could obtain enough read coverage of *SMN2* to make a quantitative statement. Although we cannot exclude contributions from the near-identical *SMNΔ7* expression construct in this analysis, sashimi plots of *SMN2* showed that there was no difference in splicing of *SMN2* across the different neuron groups (Supplemental Fig. S5C), supporting the qPCR analysis. The known SMN splicing target *Uspl1* served as a positive control for the detection of splicing differences in the LCM-seq data. Thus, the differential vulnerability of the neuron types investigated is not explained by differences in exon 7 splicing efficiency. The RNA-seq data therefore warrant further investigation to identify cell intrinsic mechanisms of selective neuronal vulnerability in SMA.

### SMA mice do not present a general developmental delay as determined by gene expression and neuromuscular junction analyses

**Figure 2.**
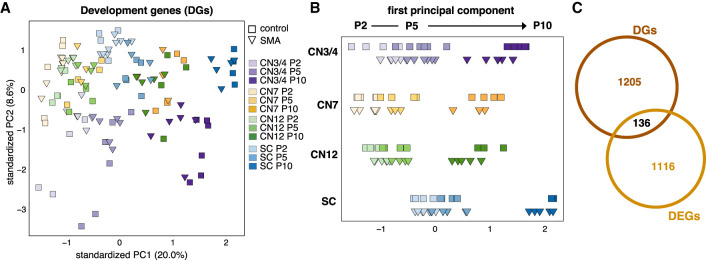
Evaluation of the developmental state of SMA somatic motor neuron somas. (*A*) PCA with development-related genes (DGs, 1341 genes) of all somatic motor neurons in control and SMA. (*B*) Plot of PC1 alone to better visualize the “age component.” (*C*) Venn diagram depicting overlap between DGs and all differentially expressed genes between control and SMA (DEGs, no fold-change cutoff, *P*_adj_ < 0.05) per cell type and time point.

It has been described that SMA patients and transgenic mouse models display a developmental delay in their neuromuscular systems (for review, see Hausmanowa-Petrusewicz and Vrbová 2005). Differences in the developmental state of SMA and control motor neurons that may not reflect a pathological mechanism per se could hamper the identification of disease-relevant transcriptional changes. We thus addressed this issue here using the gene expression data. We first used weighted gene correlation network analysis (WGCNA) to identify gene sets that were regulated during early postnatal development of control somatic motor neurons. We chose the three modules that were most highly correlated with the early (P2) and late (P10) time points and changed over time (from a negative to a positive correlation or vice versa) (Supplemental Fig. S6A,B; Supplemental Table S2). PCA with a total of 5843 genes in these modules confirmed the separation of somatic motor neurons based on age along the first principal component (PC1) (Supplemental Fig. S6C), supporting a change in expression of these genes during normal postnatal development. Gene Ontology (GO) analysis revealed enrichment of several terms related to development (Supplemental Fig. S6D), and we used the 1341 genes belonging to these terms (Supplemental Table S2) to perform PCA of control and SMA samples. As expected, the PCA showed a clear age component between P2 and P10 samples. However, SMA samples did not appear to cluster differently on the age axis (PC1) compared to control samples (Fig. 2A). To better visualize the position of the SMA samples along the “age component,” we plotted each cell type separately along PC1. We did not observe any difference in the age component in P2 and P5 samples but P10 SMA samples shifted toward the younger age (PC1 negative) relative to control samples (Fig. 2B). Because this shift occurred only at a time when SMA mice are visibly affected by disease, it likely reflects a disease process rather than a general developmental delay. In support of this, only 10.1% (136 genes) of all developmentally regulated genes were also significantly differentially expressed in disease (Fig. 2C; Supplemental Table S2), confirming that the majority of age-regulated genes are not affected in SMA. Further, if there was indeed a developmental delay in SMA motor neurons, we would expect to find genes regulated in the opposite directions in development and disease. However, of the 71 genes with opposite regulation, 76% (54 genes) were only regulated at the latest time point in disease, supporting our findings from the PCA (Fig. 2A,B). Altogether, our data challenge the notion that SMA motor neuron somas display a general developmental delay.

To exclude any possible peripheral developmental phenotype in the neuromuscular system in SMA mice, we quantified the levels of poly-innervation and endplate perforations in target muscle groups. These measures are excellent indicators of early postnatal developmental stages until P14 in mice. Our analysis of extraocular muscles (EOM; innervated by CN3/4 motor neurons), tongue muscles (innervated by CN12 motor neurons), and lumbrical muscles from the hind limbs (innervated by lumbar SC motor neurons) showed that the level of poly-innervation was equal in SMA mice and control littermates (multiple two-tailed *t*-tests) (Supplemental Fig. S7A; Supplemental Table S3). We could also show that perforations were completely unaffected by disease in tongue muscle, and only very slightly affected in extraocular muscles at end-stage of disease (P14, multiple two-tailed *t*-tests, *P*_adj_ < 0.05). As expected, lumbrical muscle endplates were severely affected at late stages of disease, lacking in perforations compared to control muscles (multiple two-tailed *t*-tests, *P*_adj_(P10) < 0.05, *P*_adj_(P14) < 0.0001) (Supplemental Fig. S7B; Supplemental Table S4). Collectively, the NMJ poly-innervation and endplate perforation data show that there was no obvious developmental delay in the maturation of neuromuscular synapses in agreement with the transcriptome data. We therefore conclude that there is no major developmental delay in the motor system of the SMA mouse model, but that it is affected as disease progresses. Consequently, the same ages in control and SMA mice can be compared to distinguish disease-induced transcriptional changes without developmental processes obscuring the data sets.

### SMA-induced gene expression changes imply a common TRP53-mediated stress response in vulnerable and resistant somatic motor neurons

**Figure 3.**
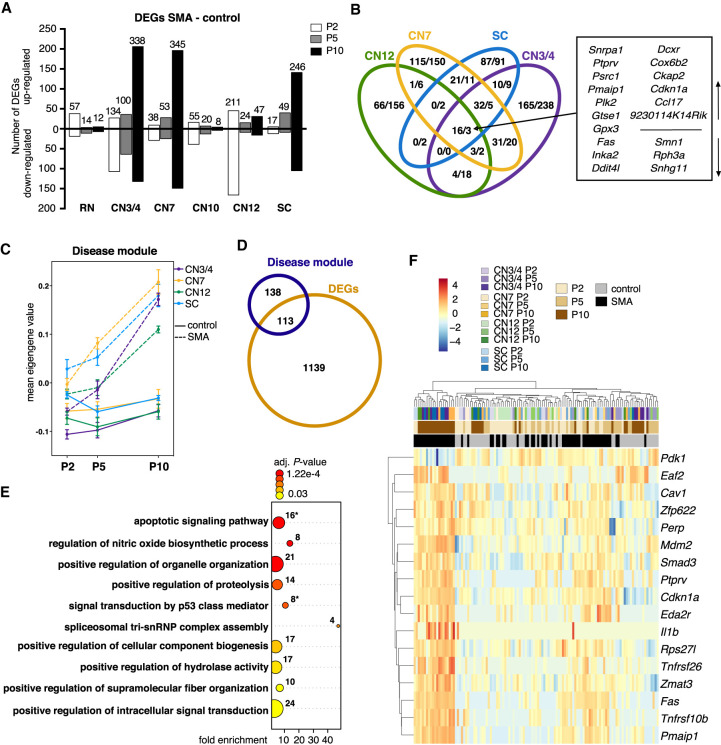
Analysis of disease-induced gene expression changes in SMA somatic motor neurons. (*A*) Number of significant genes from pairwise differential expression analysis per cell type and time point (DEGs, no fold-change cutoff, *P*_adj_ < 0.05). Numbers on bars represent total numbers of DEGs. (*B*) Venn diagram depicting the overlap in gene expression changes between somatic motor neurons (number of up-/down-regulated genes in SMA, all time points combined). (*C*) Mean eigengene values (first principal component of the disease module) within replicates. (*D*) Venn diagram depicting the overlap between genes in the disease module and DEGs. (*E*) GO term analysis for biological processes of the 251 genes in the disease module. Shown are selected GO terms; a complete list of enriched terms can be found in Supplemental Table S9. Numbers indicate the number of genes in a given term, color scale is the adjusted *P*-value. Asterisks indicate gene sets that are plotted in *F*. (*F*) Expression heatmap of genes that belong to GO terms related to apoptosis and TRP53 signaling. Expression values were log_2_-transformed and mean centered.

Toward our main goal of elucidating mechanisms of neuronal resilience and susceptibility, we investigated the transcriptional dynamics in resistant and vulnerable neurons in SMA. Using DESeq2 (Love et al. 2014), we performed pairwise differential expression analyses between control and SMA per cell type and time point. We found the strongest early transcriptional response in resistant CN3/4 and CN12 motor neurons with 134 and 211 differentially expressed genes (DEGs; no fold-change cutoff, adjusted *P*-value <0.05) at P2, followed by relatively resistant RN and CN10 neurons with 57 and 55 regulated genes, respectively. At the late disease stage (P10), CN3/4, CN7 and SC motor neurons showed a large number of disease-regulated genes, and RN and CN10 neurons showed minimal gene expression changes (Fig. 3A; Supplemental Table S5). Hierarchical clustering using DEGs of all neuronal populations separated SMA samples from controls at the earliest disease stage analyzed (P2) (Supplemental Fig. S8A). At later disease stages (P5–P10) somatic motor neuron groups clearly clustered together based on genotype, but RN and CN10 neurons did not separate with disease (Supplemental Fig. S8B,C). This shows that resistant CN3/4 motor neurons display a response to disease that is distinct from other relatively resilient neuron groups.

To identify uniquely and/or commonly regulated genes across somatic motor neuron groups, we plotted all DEGs in a Venn diagram. We only identified three commonly down-regulated genes across all somatic motor neurons: *Smn1*, *Rph3a*, and *Snhg11*. Sixteen transcripts were commonly up-regulated with disease, including *Snrpa1, Plk2, Inka2, Ddit4l, Dcxr, Cox6b2, Ccl17*, *9230114K14Rik, Ptprv, Psrc1, Pmaip1, Gtse1, Gpx3, Fas, Ckap2*, and *Cdkn1a* (Fig. 3B). The majority of gene expression changes, however, were unique to each neuron group (Fig. 3B; Supplemental Fig. S8D; Supplemental Tables S6, S7), indicating distinct mechanisms to adapt to the loss of *Smn*. To further control for cell type–driven differences, we performed differential gene expression analysis between CN3/4 and the other somatic groups and selected genes that were only highly significant with disease (Supplemental Fig. S9). The minor overlap found here is in support of cell type–specific transcriptional changes in SMA.

To corroborate our DESeq2 analysis, we conducted WGCNA for all somatic motor neurons (both control and SMA samples), which revealed a module consisting of 251 genes that was highly positively correlated with disease and negatively correlated with control samples (Supplemental Table S8), which we will refer to as “disease module.” As shown by the mean eigengene values for the module within sample replicates, there was a clear genotype separation with disease progression in all somatic populations independent of their vulnerability (Fig. 3C). We plotted all genes of the module in a heatmap, which confirmed the similar expression levels across all somatic motor neurons (Supplemental Fig. S8E). Conversely, the relatively unaffected RN and CN10 neurons did not show separation based on disease and displayed unique responses to disease (Supplemental Fig. S8F). Besides *Smn*, only one gene of the disease module, *Plek2*, was differentially expressed in RN, and four genes, *Lars2, Olig2, Ptgds*, and *Ubap1l*, were regulated in CN10 motor neurons with disease (Supplemental Fig. S8G). Furthermore, 45% of the genes (113 genes) in the module were identified as significantly differentially expressed with disease in one or more somatic populations using DESeq2 (Fig. 3D), including 14 of the 19 DEGs that are common to all somatic motor neurons. We thus identified a disease signature specific to somatic motor neurons independent of their vulnerability. GO analysis for biological processes resulted in the significant (adjusted *P*-value < 0.05) enrichment of 23 GO terms in total (Fig. 3E; Supplemental Table S9). The pathways we identified as regulated included, for example, apoptotic signaling, signal transduction induced by p53 class mediator, positive regulation of intracellular signal transduction, spliceosomal tri-snRNP complex assembly, and several terms suggesting changes in metabolism (proteolysis, hydrolase activity) as well as cellular component organization (e.g., organelle organization, supramolecular fiber organization, cellular component biogenesis). To better visualize the timing of TRP53 and apoptosis marker expression in somatic motor neurons, we plotted the 17 dysregulated genes identified in these pathways in a heatmap (Fig. 3F). This analysis clearly showed that activation of this pathway strengthened with disease progression and was shared by all somatic motor neuron populations investigated here. Consistently, GO term analysis of DEGs indicated a TRP53 pathway activation in SMA SC motor neurons already at P5 and confirmed the regulation of TRP53 and DNA damage pathways at P10 in SC, CN7, and CN3/4 motor neurons (Supplemental Table S9). In contrast, GO term analysis of DEGs in RN and CN10 did not yield any statistically significant results. Furthermore, eight of the 16 commonly up-regulated genes from the DESeq2 analysis (Fig. 3B) are involved in the regulation of cell death, stress, and/or TRP53 signaling. We used the STRING database to retrieve protein–protein interaction networks within the disease module. As expected, the network confirmed a coordinated TRP53-pathway activation. We further revealed a subnetwork of genes involved in RNA processing and splicing, consistent with the role of SMN in spliceosome assembly (Supplemental Fig. S10).

In summary, we revealed a potentially detrimental common disease signature in all somatic motor neurons that is absent in RN and CN10 neurons. Ocular motor neurons presented a unique adaptation mechanism to the loss of *Smn* that warranted further investigation.

### Resistant motor neurons activate a unique transcriptional program that could confer protection in SMA

**Figure 4.**
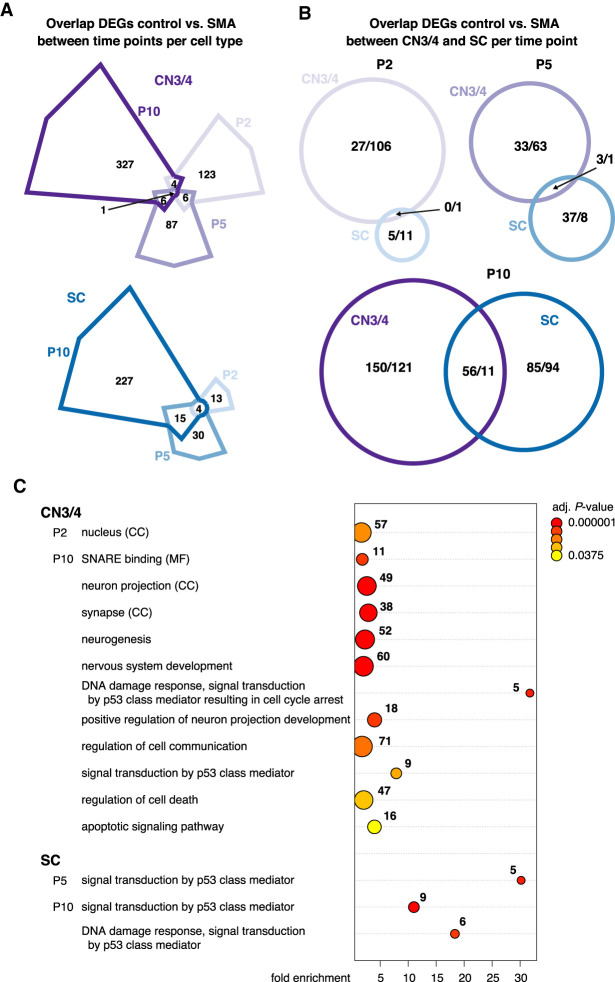
Comparison of gene expression changes in ocular and spinal motor neurons. (*A*) Venn diagrams depicting shared and time point–specific DEGs between control and SMA motor neurons in CN3/4 (*top*) and SC (*bottom*). (*B*) Venn diagrams illustrating the overlap of DEGs between CN3/4 and SC at each time point. (*C*) GO term analysis of DEGs in CN3/4 and SC per time point. Shown are selected terms; a complete list of enriched terms can be found in Supplemental Table S9. Numbers indicate the number of genes in a given term, and the color scale shows the adjusted *P*-value. Terms belong to the domain biological processes unless specified otherwise: (CC) cellular compartment; (MF) molecular function.

To understand why CN3/4 motor neurons remain resilient to degeneration in SMA while apoptotic signaling pathways appear activated, we conducted a close comparison with vulnerable SC motor neurons. Both neuron groups displayed distinct temporal responses (Fig. 4A; Supplemental Table S10). There was no overlap between CN3/4 and SC motor neurons in their early response to loss of *Smn* (Fig. 4B; Supplemental Table S11). Forty-three percent of all CN3/4-regulated genes at P2 belong to the GO term “nucleus,” including several genes involved in RNA processing and transcriptional activation/repression such as *Cnot9, Ube2b, Wtap, Cbx6, Hdac6, Ino80*, and *Jmjd1c* (Supplemental Table S9), suggesting an early fine-tuning of gene expression. As disease progressed, more genes were jointly regulated across the neuron groups but the majority of transcriptional changes were still unique to each neuron type. Specifically, at P5, three genes were commonly up-regulated in CN3/4 and SC motor neurons; at P10, 56 genes were commonly up-regulated and 11 down-regulated (Fig. 4B). Thus, 100% of the genes regulated at P2 were unique to CN3/4 motor neurons, 97% at P5, and 80% at P10.

**Figure 5.**
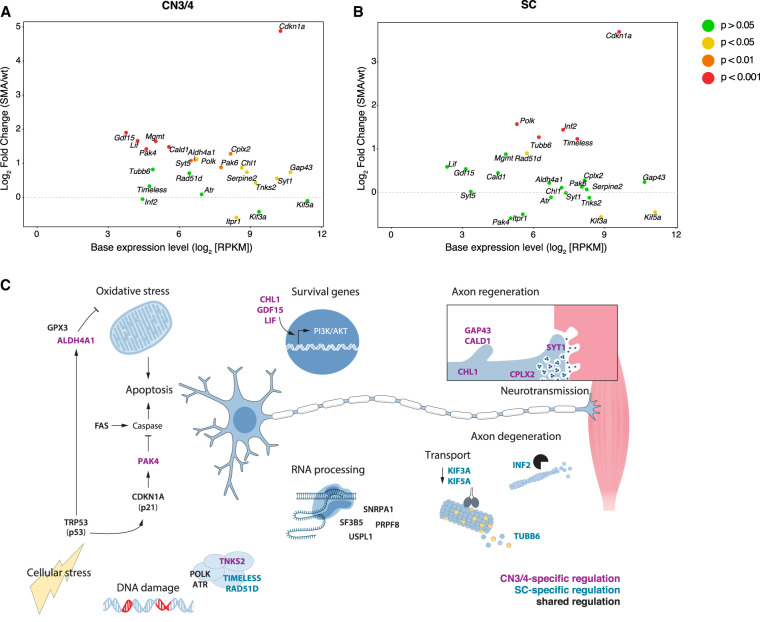
Common and cell type–specific disease mechanisms in SMA. Expression changes in key genes between SMA and wild type in CN3/4 (*A*) and SC motor neurons (*B*). Colors indicate significance levels. (*C*) Somatic motor neurons display transcriptional changes caused by the loss of full-length SMN protein that are distinct from red nucleus and vagus (CN10) neurons. For example, prominent changes in expression levels of genes that function in RNA processing are restricted to somatic motor neurons. These neurons are furthermore exposed to cellular stress, including oxidative stress and DNA damage, and DNA repair genes are induced. TRP53- and cell death signaling pathways are activated in all somatic motor neurons independent of their susceptibility to degeneration in SMA. Vulnerable spinal (SC) motor neurons show signs of axon degeneration and axonal transport deficits. Resistant ocular (CN3/4) motor neurons selectively up-regulate the expression of genes that counteract apoptosis and promote cell survival. Increased levels of genes functioning in neurite outgrowth, axon regeneration, and neurotransmission, which support the maintenance of a functional neuromuscular synapse, are also seen in ocular motor neurons in disease.

GO term analysis of DEGs in CN3/4 neurons at P10 pinpointed a number of fundamental processes that were activated in response to disease. These pathways included neurogenesis, nervous system development, positive regulation of neuron projection development, regulation of cell communication, regulation of apoptotic processes, and cell death (Fig. 4C; Supplemental Table S9). Among the enriched cellular compartments were neuron projection and synapse. To visualize CN3/4-restricted pathways that could be protective, we used the STRING database to retrieve protein–protein interaction networks from all DEGs at P10. We obtained a highly interconnected protein network that consisted of 158 genes, corresponding to 46.7% of all DEGs in CN3/4 at P10 (Supplemental Fig. S11), thus indicating a highly coordinated transcriptional response. The respective network for DEGs in SC motor neurons consisted of only 61 genes (25.5% of all DEGs) and a second major network included 22 genes (8.9% of all DEGs) (Supplemental Fig. S12). TRP53 and 15 of the directly interacting proteins were up-regulated in both neuron types (Supplemental Figs. S11, S12, gray outlines), in line with the GO term analysis. Up-regulated genes involved in DNA damage repair, were *Polk* (shared), *Tnks2* and *Mgmt* (CN3/4-specific), and *Rad51d* and *Timeless* (SC-specific). CN3/4-specific down-regulation included pro-apoptotic factors like *Itpr1* and *Dffa*, which was accompanied by the up-regulation of anti-apoptotic and survival factors such as *Pak4, Pak6, Chl1, Tmbim4, Aldh4a1*, and *Gdf15.* Neurotransmitter release is impaired in motor nerve terminals in SMA (Ruiz and Tabares 2014). It is therefore compelling to see the CN3/4-specific regulation of genes involved in neurotransmitter release, including the up-regulation of *Syt1, Syt5*, and *Cplx2*, suggesting a compensatory mechanism in the disease-resistant cells. Among the many DEGs that are implicated in cytoskeletal reorganization, we found CN3/4-specific regulation of genes that are important for neurite outgrowth including *Gap43, Chl1, Syt1, Cald1*, and *Serpine2.* (Fig. 5A,C; Supplemental Fig. S11).

In contrast, in vulnerable SC motor neurons, we found increased levels of *Inf2*, which can disassemble actin filaments (Supplemental Table S5), and the tubulin isoform *Tubb6*, which is associated with decreased microtubule stability (Bhattacharya and Cabral 2004; Salinas et al. 2014). We also found a significant decrease in mRNA levels of several motor proteins (*Dnahc, Kif3a, Kif5a*) including genes that function in the anterograde transport of a variety of cargos to the cell periphery including the synapse (Fig. 5B,C; Supplemental Fig. S12).

Thus, we identified multiple transcriptional programs specifically activated in ocular motor neurons in SMA that could confer protection against detrimental disease processes.

### The oculomotor-regulated factor GDF15 confers protection onto human spinal motor neurons

**Figure 6.**
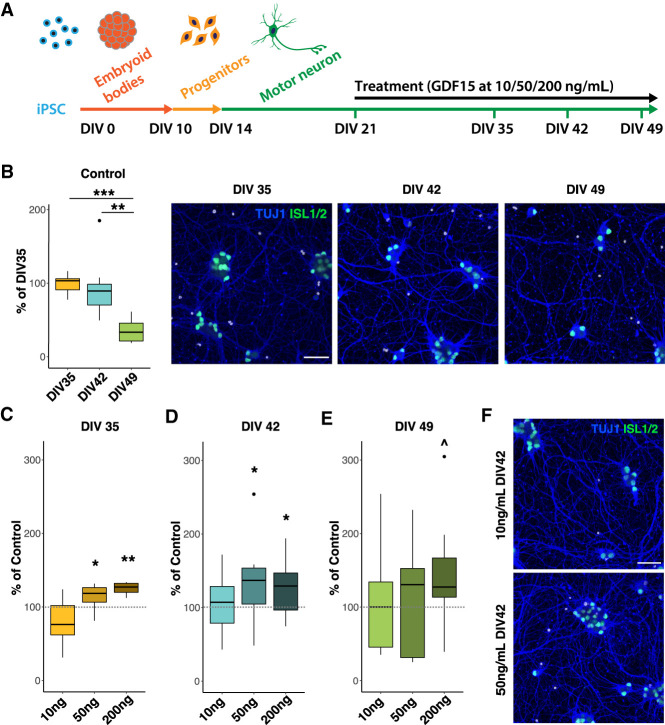
The oculomotor-enriched factor GDF15 protects human spinal motor neurons. (*A*) Schematic of the differentiation protocol for human iPSCs into motor neurons and the treatment with GDF15. (*B*) Human spinal motor neurons degenerate over time in culture when grown without growth factors. Representative images show immunostaining with antibodies against ISLET-1/2 (ISL1/2) and ßIII-tubulin (TUJ1). (*C*) At day 35 in vitro (DIV35), addition of 50 or 200 ng/mL GDF15 improves survival of motor neurons compared to control, but 10 ng/mL had no effect. (*D*) At DIV 42, 50 and 200 ng/mL GDF15 protects motor neurons. (*E*) At DIV 49, there is no significant effect of GDF15 at concentrations 10–200 ng/mL. (*F*) Representative images showing motor neurons treated with 10 ng/mL or 50 ng/mL GDF15 at DIV42. The scale bar in *B* and *F* is 50 µM. Student's *t*-test: (***) *P* < 0.001; (**) *P* < 0.01; (*) *P* < 0.05; (^) *P* < 0.1.

Next, we wanted to confirm our hypothesis that adaptation mechanisms identified in resilient neurons can be used to reduce susceptibility of vulnerable motor neurons. We thus analyzed the effect of adding GDF15, which was highly up-regulated uniquely in resilient CN3/4 motor neurons in SMA, onto vulnerable spinal motor neurons. For this purpose, we generated human spinal motor neurons from induced pluripotent stem cells (iPSCs) according to established protocols (Fig. 6A; Guo et al. 2017; Nijssen et al. 2019). We first showed that these human neurons degenerate over time in culture in a growth factor–deprivation assay (Fig. 6B; Lamas et al. 2014). We then showed that addition of GDF15 to these vulnerable spinal motor neurons significantly improved their survival in a dose-dependent manner (Fig. 6C–F). The effect was evident when the cell death assay was not too harsh, days 35–42 in vitro (Fig. 6C,D,F). This is clear confirmation that targets identified in resilient neurons using our approach can be used to reduce susceptibility of vulnerable motor neurons.

Collectively, our analysis shows that resistant neurons respond early and uniquely to the loss of *Smn.* Arguably this response involves the regulation of bona fide neuroprotective factors and processes, as exemplified by *Gdf15* that was uniquely up-regulated in CN3/4 motor neurons with disease and could protect vulnerable spinal motor neurons from degeneration when added to these.

## Discussion

In this study, we conducted a longitudinal analysis of the transcriptional dynamics in CN3/4 (ocular), CN7 (facial), CN10 (vagus), CN12 (hypoglossal), SC (spinal), and RN (red nucleus) neuron groups, which show differential vulnerabilities to degeneration during disease progression in SMA mice. We found that, independent of their vulnerability, somatic motor neurons activate TRP53 signaling pathways that are associated with DNA damage and cell death. This up-regulation was absent in visceral vagus motor neurons and red nucleus neurons. This suggests that the activation of the TRP53 pathway is a stress response specific to somatic motor neurons in SMA but that it does not necessarily lead to degeneration as ocular motor neurons persist. We also show that the majority of gene expression changes induced by the loss of *Smn* are cell type–specific and reveal several pathways that are restricted to resistant ocular motor neurons. Such bona fide protective pathways include increased levels of survival factors, and pro-apoptotic genes are selectively down-regulated (Fig. 5A–C). We also observed ocular motor neuron-restricted transcriptional regulation of genes involved in neurotransmission and neurite outgrowth that may aid in the maintenance of a functional neuromuscular synapse and thus contribute to the selective resistance of these motor neurons in SMA.

TRP53, which was up-regulated across all somatic motor neuron groups in SMA, is a master regulator in response to several cellular stressors such as oxidative stress or DNA damage. SMN directly interacts with TRP53 (Young et al. 2002), and transcriptional activation of *Trp53* or its target genes has been previously shown in different models of SMA (Zhang et al. 2008, 2013; Bäumer et al. 2009; Murray et al. 2015; Staropoli et al. 2015; Jangi et al. 2017; Simon et al. 2017). Cell death signaling was not restricted to vulnerable populations in our study, but was also evident in resistant neurons, predominantly at a late stage of disease (P10). Consistently, more resistant spinal motor neurons of the lateral motor column show increased TRP53 protein levels with progression of disease (Simon et al. 2017). TRP53 itself can activate a number of targets that exert anti-apoptotic effects, such as *Gtse1*, *Dcxr*, *Gpx*, and *Cdkn1a* (for review, see Jänicke et al. 2008), which were also up-regulated in all the somatic populations. Specifically, the cyclin-dependent kinase inhibitor 1A (P21) (*Cdkn1a*) plays a crucial role in cell cycle regulation and response to DNA damage. *Cdkn1a* can also prevent apoptosis, for instance, by transcriptional repression of pro-apoptotic genes or inhibiting caspases (for review, see Gartel and Tyner 2002). Thus, the up-regulation of *Cdkn1a* could be part of a protective response in somatic motor neurons, which is not sufficient to protect the most vulnerable cells. Apparent shared protective responses may also in part stem from the enrichment of more resilient neurons within the vulnerable populations with time, for example, enrichment of the more resilient lateral spinal motor neurons versus more medial motor neurons (Mentis et al. 2011) and CN7 neurons innervating the rostral versus caudal band of the levator auris longus muscle (Murray et al. 2008). Back-labeling techniques, as used by Murray et al. (2015), or single-cell RNA sequencing (Nichterwitz et al. 2016; Hedlund and Deng 2018) could further aid in the distinction of vulnerable and resistant cells within neuronal populations to further dissect protective from detrimental pathways. However, even at P10, somas of vulnerable motor neurons that show peripheral pathology (Comley et al. 2016) are still present in the spinal cord. Thus, although we cannot exclude that some detrimental pathways were masked by the inclusion of more resilient neurons, we are confident that we have included vulnerable cells at all time points.

With our comprehensive comparison of resistant ocular and vulnerable spinal motor neurons using Gene Ontology and protein network analyses, we were able to pinpoint several protective pathways that are selectively regulated in the resilient motor neuron group and thus likely counteract commonly activated stress responses. These are pathways that appear particularly compelling to modulate in susceptible neurons to make these more resilient to degeneration. Prosurvival factors with increased levels in ocular motor neurons in SMA were, for instance, (1) the growth differentiation factor 15 (*Gdf15*), which can protect dopamine neurons from Parkinson-like 6-hydroxydopamine-induced degeneration (Strelau et al. 2000). GDF15-deficient mice show a progressive loss of motor neurons in spinal cord and several brain stem nuclei, suggesting that it is a genuine trophic factor for motor neurons (Strelau et al. 2009). (2) Leukemia inhibitory factor (LIF) promotes survival of primary rat and mouse motor neurons grown in vitro (Martinou et al. 1992; Arce et al. 1999). *Lif* knockout mice display loss of distal motor axons and decrease in motor endplate size, indicating that it also has an important role for maintaining motor neuron connectivity with muscle (Holtmann et al. 2005). (3) The mitochondrial aldehyde dehydrogenase ALDH4A1 can safeguard cells from oxidative stress (Yoon et al. 2004). There is recent evidence for increased oxidative stress linked to mitochondrial dysfunction in primary motor neuron cultures from SMA mice (Miller et al. 2016). (4) CDKN1A activated kinases PAK6 and PAK4; PAK4 prevents caspase activation and thus apoptosis (Gnesutta et al. 2001; Gnesutta and Minden 2003) and its neuroprotective function was recently shown in a rat model of Parkinson's disease (Won et al. 2016). (5) TMBIM4, also known as Lifeguard4, shows anti-apoptotic functions likely through modulation of intracellular Ca^2+^ (for review, see Carrara et al. 2017). Consistently, the Ca^2+^ channel *Itpr1* (IP3 Receptor), which plays a role in endoplasmic reticulum (ER) stress-induced apoptosis, was selectively down-regulated in ocular motor neurons. The activation of ER stress pathways in SMA was shown in a transcriptomics study using iPSC-derived motor neurons from SMA patients (Ng et al. 2015). Thus, resistant ocular motor neurons appear to, by necessity, regulate pathways that counteract the detrimental processes that are activated with disease in all somatic motor neurons. Other resistant neurons groups, including red nucleus and visceral motor neurons showed no such compensatory gene regulation, but also lacked DNA damage pathway and TRP53 activation.

As SMN also functions locally in the axon, including in nerve terminals, preventing apoptosis is unlikely to fully rescue motor neuron function. We therefore believe that genes involved in neurotransmission and neurite outgrowth, that would affect neighboring neurons and muscle in addition to motor neurons themselves, are important candidates for neuroprotection. Exciting candidates that were specifically up-regulated in ocular motor neurons with disease were synaptotagmin 1 and 5 (*Syt1* and *Syt5*) (Fig. 5A,C). SYT1 functions in the release of synaptic vesicles and has recently been associated with differential vulnerability in SMA (Tejero et al. 2016). By counteracting the impaired neurotransmitter release that has been observed in SMA motor neurons (Kariya et al. 2008; Ruiz and Tabares 2014), ocular motor neurons may be able to maintain their connection to target muscles and ensure their functionality. In support of this, we also found complexin II (*Cplx2*) up-regulated in ocular motor neurons, which also modulates synaptic vesicle release (Ono et al. 1998). The genetic depletion of *Cplx2* in mice results in locomotor deficits (Glynn et al. 2003), suggesting an important function in motor neurons. We also identified caldesmon 1 (*Cald1*), the growth associated protein 43 (*Gap43*), and the L1 cell adhesion molecule homolog *Chl1* to be selectively up-regulated in ocular motor neurons. CALD1 is a regulator of neurite outgrowth (Morita et al. 2012), and GAP43 is crucial for developmental axon outgrowth and regeneration. Likewise, CHL1 levels increase in regenerating motor neurons after sciatic nerve injury (Zhang et al. 2000); it is a survival factor for primary rat motor neurons, acting via the PI3K/AKT pathway (Nishimune et al. 2005). It was recently shown that *Gap43* mRNA and protein levels were reduced in axons and growth cones of primary spinal motor neurons isolated from a severe mouse model of SMA (Fallini et al. 2016), predisposing these to a lower degree of reconnectivity. Thus, the SMA-induced increase in *Cald1, Gap43*, and *Chl1* in ocular motor neurons could help these to reconnect to muscle targets if disconnected during disease.

Furthermore, our functional analysis supports the idea that an up-regulation of prosurvival factors could in part account for neuronal resilience as the oculomotor-regulated factor GDF15 protected vulnerable human spinal motor neurons from degeneration. The protective effect was most prominent during the first 21 d of the growth-deprivation assay (up until day 42). Thereafter, the assay becomes very harsh with a 58% drop in motor neurons within a 7-d period, and a rescue is thus much more difficult to orchestrate. The neuroprotection seen is very encouraging because it shows that factors up-regulated within ocular motor neurons with disease are protective and that using the response of resilient neurons to disease can identify pathways that can be used to protect also vulnerable neurons. Although GDF15 alone can support motor neuron survival for an extended amount of time, we believe that treatment with a combination of factors up-regulated within ocular motor neurons will be even more beneficial for motor neuron survival. Future studies will therefore aim to determine the most optimal cocktail of ocular motor-regulated factors through gain- and loss-of-function studies.

In summary, the transcriptional regulation of genes related to neuroprotection, neurotransmission, and neurite outgrowth presents compelling bona fide protective mechanisms that are activated during disease progression selectively in these resistant motor neurons.

In conclusion, this study provides important insights into mechanisms of selective resistance and vulnerability in SMA. We show that all somatic motor neurons, irrespective of their vulnerability in SMA, present stress responses owing to SMN deficiency. However, resistant ocular motor neurons selectively activate survival pathways, including *Gdf15, Lif*, and *Chl1*, which could protect vulnerable spinal motor neurons from degeneration, and show transcriptional regulation of genes that are important for the maintenance and/or regeneration of a functional neuromuscular synapse. The modulation of such mechanisms presents a promising strategy, not only for the additional treatment of SMA patients in which splicing correction of *SMN2* is not sufficient but also other motor neuron diseases like ALS. We thus revealed novel targets that will be exciting to investigate further, both alone and in combinations, in the context of motor neuron disease.

## Methods

### Ethics statement and animal model

All work was carried out in accordance with the Code of Ethics of the World Medical Association (Declaration of Helsinki) and with national legislation and institutional guidelines. Animal procedures were approved by the Swedish animal ethics review board (Stockholm Norra Djurförsöksetiska Nämnd). Ethical approval for the use of human iPSCs was obtained from the regional ethical review board in Stockholm, Sweden (Regionala Etikprövningsnämnden, Stockholm, EPN).

Animals were housed under standard conditions with a 12-h dark/light cycle and had access to food and water ad libitum. Neonatal pups were used as a model of SMA (*Smn*^−/−^/*SMN2*^+/+^/*SMNΔ7*^+/+^), and age matched littermates that were homozygously wild type for murine *Smn* (*Smn*^+/+^/*SMN2*^+/+^/*SMNΔ7*^+/+^) were used as controls (Le et al. 2005) (Jackson Laboratory stock number 005025). For transcriptomics, we used 2-, 5-, and 10-d old pups (P2, P5, and P10), whereas neuromuscular junction analysis was performed on 5-, 10-, and 14-d old pups (P5, P10, and P14). P2 and P5 pups were sacrificed by decapitation, and P10 and P14 pups were anesthetized with a lethal dose of avertin (2,2,2- Tribromoethanol in 2-Methylbutanol, Sigma-Aldrich) before decapitation.

### Laser capture microdissection of distinct neuronal populations

Six neuronal populations (CN3/4, RN, CN7, CN10, CN12, spinal motor neurons) were collected using laser capture microdissection, as previously described (Nichterwitz et al. 2016, 2018). Brain and spinal cord tissues were dissected and immediately snap frozen. Coronal cryosections (12 µm) were prepared and placed onto PEN membrane glass slides (Zeiss). Immediately before LCM, a quick histological staining was performed to visualize cells (Histogene, Arcturus). After 100–200 cells were collected into the dry cap of a PCR tube, 5 µL of lysis buffer was added and samples were snap frozen on dry ice. For a more detailed description, see Supplemental Methods.

### cDNA and sequencing library preparation

Library preparation was performed with a modified version of the Smart-seq2 protocol (Picelli et al. 2013, 2014) and was previously described in detail (Nichterwitz et al. 2016, 2018; see also Supplemental Methods). Equal amounts of cDNA from up to 30 samples were pooled per lane of a flow cell. Then, 43-bp single-end sequencing was performed on an Illumina HiSeq 2000 sequencing platform resulting in an average read depth of 14.1 ± 0.4M (mean ± SEM) reads per sample.

### RNA-seq data analysis

The RNA-seq reads were mapped simultaneously to the mm10 mouse genome assembly and the genomic sequence of human *SMN2* (including introns) to the hg19 assembly using STAR (version 2.4.1c) (Dobin et al. 2013). The genomic sequence of *SMN2* is identical between the hg19 and hg38 human genome versions; thus, aligning the reads to either genome would give identical results. Quality control was performed with rrnaseq (https://github.com/edsgard) to ensure sufficient sequencing depth and mapping ratios appropriate for this poly(A)-enriched sequencing strategy. We used uniquely mapped reads (69.1 ± 0.39% mean ± SEM) (Supplemental Fig. S3B) for further analyses. Expression levels were determined using the rpkmforgenes.py software (http://sandberg.cmb.ki.se/rnaseq) with the Ensembl gene annotation. Only samples with more than 11,000 detected genes (≥1 RPKM) were included in the analysis. A detailed description of principal component analyses (PCAs), weighted gene correlation network analysis (WGCNA), differential gene expression, GO term, and STRING analyses is supplied in Supplemental Methods.

### Use of published data sets

Some samples used in this study (control SC P5 samples) were previously deposited into GEO by our laboratory with the accession number GSE76514. For the evaluation of the purity of the samples, we compared them to a previously published data set (Zhang et al. 2014). Raw data were obtained from the Gene Expression Omnibus (GEO, accession number GSE52564) and processed as described for our own samples.

### Growth factor–deprivation assay on human iPSC-derived motor neurons

For derivation of motor neurons, an established differentiation protocol was used (Guo et al. 2017; Nijssen et al. 2019). After dissociation of embryoid bodies at day 10, motor neuron progenitors were plated in clear bottom, black 96-well plates (CLS3603, Corning), coated with laminin (Sigma-Aldrich), Fibronectin (Sigma-Aldrich), and poly-L-ornithine (Thermo Fisher Scientific), at 17,000 cells/well in Neurobasal supplemented with B27, 10 ng/mL of both brain-derived neurotrophic factor (BDNF, Peprotech) and glial cell-derived neurotrophic factor (GDNF, Peprotech), 200 nM retinoic acid (Sigma-Aldrich) for 1 d, and 10 μM DAPT (Tocris) for 4 d. After day 14, the media consequently contained only BDNF, GDNF, and ascorbic acid; media was changed every other day. For the growth factor deprivation, BDNF and GDNF were removed from the media at day 21. From this time point and onward, the motor neurons were treated with GDF15 (Peprotech) at the indicated concentrations with media changes every other day.

### Immunocytochemistry and image analysis of iPSC-derived motor neurons

Fixed motor neuron cultures were stained with mouse anti-ISL1/2 (DSHB, 39.4D5) at 1:50 and rabbit anti-Tuj1 (beta-3 tubulin, Biolegend, 802001), combined with Alexa-fluor conjugated secondary antibodies (Thermo Fisher Scientific). Nuclei were counterstained with Hoechst 33342. Two to five replicate wells were imaged per condition per experiment (12 images per well). Cells were counted using the “analyze particles” function in Fiji (ImageJ) after thresholding. The number of (Islet-positive) cells was then aggregated per well, and all data are presented as data points per well. Statistical analysis was performed using R software for statistical computing (R Core Team 2020). For the analysis of control samples, values are expressed as percent of motor neurons at DIV 35 and two-sample *t*-tests (unpaired, one-sided) were used to compare time points. For the analysis of GDF15 treatment, values are expressed as percent of motor neurons in control samples within each time point and experiment. One-sample *t*-tests were performed for each condition (GDF15 dose) against the control within each time point (mu = 100, one-sided). A detailed description can be found in Supplemental Methods.

## Data access

All raw and processed sequencing data generated in this study have been submitted to the NCBI Gene Expression Omnibus (GEO; https://www.ncbi.nlm.nih.gov/geo/) under accession number GSE115706.

## Competing interest statement

The authors declare no competing interests.

## Supplementary Material

Supplemental Material
