# Supplementary material for: LCM-seq reveals unique transcriptional adaptation mechanisms of resistant neurons and identifies protective pathways in spinal muscular atrophy
Source: Genome Res. 2020 Aug;30(8):1083–96. doi: 10.1101/gr.265017.120 (PMC7462070; doi:10.1101/gr.265017.120)
Supplement: Supplemental Material [file supp_30_8_1083__index.html]

LCM-seq reveals unique transcriptional adaptation mechanisms of resistant neurons and identifies protective pathways in spinal muscular atrophy — Supplemental Material 

# LCM-seq reveals unique transcriptional adaptation mechanisms of resistant neurons and identifies protective pathways in spinal muscular atrophy

## Supplemental Material

- Supplemental\_Table\_S2.xlsx
- Supplemental\_Table\_S5.xlsx
- Supplemental\_Table\_S6.xlsx
- Supplemental\_Table\_S7.xlsx
- Supplemental\_Table\_S8.xlsx
- Supplemental\_Table\_S9.xlsx
- Supplemental\_Table\_S10.xlsx
- Supplemental\_Table\_S11.xlsx
- Supplemental\_Material.pdf
