# Supplementary material for: LCM-seq reveals unique transcriptional adaptation mechanisms of resistant neurons and identifies protective pathways in spinal muscular atrophy
Source: Genome Res. 2020 Aug;30(8):1083–96. doi: 10.1101/gr.265017.120 (PMC7462070; doi:10.1101/gr.265017.120)
Supplement: Supplemental Material [file supp_gr.265017.120_Supplemental_Material_.pdf]

Supporting online material for:

LCM-seq reveals unique transcriptional adaptation mechanisms of resistant neurons and identifies protective pathways in spinal muscular atrophy

Nichterwitz S[1*], Nijssen J[1,4], Storvall H[2,3,4], Schweingruber C[1], Comley LH[1], Allodi I[1], van der Lee M[1], Deng Q[3,5], Sandberg R[2,3] and Hedlund E[1,6*]

** corresponding author: eva.hedlund@ki.se or eva.hedlund@dbb.su.se and susanne.nichterwitz@gmail.com*

This document includes Supplemental Methods, Supplemental Discussion, Supplemental References, Supplemental Tables S1, S3 and S4, and Supplemental Figures S1-12.

Supplemental Methods

Reagents

For all RNA-seq experiments, nuclease-free water (H_2O, LifeTechnologies) was used and all reagents were of molecular biology/PCR grade if available. Only tubes that were certified nuclease-free were used. All workbenches and equipment were cleaned with RNaseZAP (Ambion) and additionally with DNAoff (Takara) for library preparations. For the preparation of the different staining components prior to laser capture microdissection (LCM), 99.7% EtOH Aa (Solveco) and nuclease-free H_2O were used.

Laser capture microdissection of distinct neuronal populations

Brain and lumbar spinal cords were dissected and immediately snap frozen in 2-Methylbutane (Sigma-Aldrich) on dry ice. Tissue was stored at -80°C until further processing. Brains and in OCT embedded spinal cords were sectioned in a cryostat at -20°C (12 µm coronal sections) and placed onto PEN membrane glass slides (Zeiss) that were subsequently stored at -80°C until further processing. Motor neurons are easily identifiable by their large soma size and by their distinct locations in the ventral horn of the spinal cord and in the brainstem. Tissue was therefore subjected to a quick histological staining (Histogene, Arcturus/LifeTechnologies). Immediately prior to staining, slides were thawed for 30 seconds and subsequently placed into 75% EtOH for 30 seconds. After incubation for another 30 seconds in H_2O, slides were incubated with 150-200 µl of Histogene staining solution for 20 seconds. Staining solution was removed by tapping the slide on lint-free tissue, followed by incubation for 30 seconds in H_2O. Dehydration was achieved by incubating the slides in EtOH solutions of rising concentration (75%, 95% and 99.7% EtOH, 30 seconds each). Slides were then placed into the slide holder of the microscope and cells were captured using the Leica LMD7000 system. LCM was performed at 40x magnification and cutting outlines were drawn in close proximity to individual cells to minimize contamination by surrounding tissue. Only cells with an area of more than 200 μm^2 (150 μm^2 for vagus motor neurons) and a visible nucleus with nucleolus were selected. 100-200 cells were collected into the dry cap of 0.2 ml PCR soft tubes (Biozym Scientific). 5 µl lysis buffer (0.2% Triton X-100, with 2 U/µl recombinant RNase inhibitor, Clontech) were added to

the cap and mixed by pipetting up and down 5 to 10 times. Samples were spun down using a table centrifuge (VWR) for 10 seconds and snap frozen on dry ice. The duration from thawing the slides until freezing of the sample never exceeded 2 hours. Samples were stored at -80°C until further processing.

cDNA and sequencing library preparation

For reverse transcription (RT), polymerase chain reaction (PCR) cycles and heat incubation steps a T100 Thermal Cycler was used (BioRad). RT was followed by 18 cycles of PCR amplification. After purification with magnetic beads (GE Healthcare), cDNA concentration and quality of cDNA libraries were determined with an Agilent 2100Bioanalyzer using the High Sensitivity DNA kit. For the generation of sequencing libraries, 1 ng of cDNA was used (as determined with Bioanalyzer, 100-9000 bp range). The tagmentation reaction was carried out with 0.4 -1 µl of in house Tn5 (Picelli et al. 2014a). Ligation of sequencing indices (Nextera XT Sequencing Index Kit, Illumina) and enrichment PCR (10 cycles) was performed with Kapa HiFi polymerase. Before pooling the sequencing libraries, a purification step with magnetic beads was performed and the concentration of each sample was determined on a Qubit fluorometer (Thermo Fisher Scientific) with the dsDNA high sensitivity kit (LifeTechnologies). An equal amount of cDNA from up to 30 samples was pooled per lane of a flow cell. Single read 43-bp sequencing was performed on an Illumina HiSeq 2000 sequencing system. Samples that were re-sequenced and contain two raw data (.fastq) files were pooled during the mapping procedure.

***SMN2* splicing analysis**

For real-time quantitative PCR, 0.5 ng of Smart-seq2 libraries were used per 15 µL reaction with 500 nM of each assay primer (SMN2 total, 5'-GTG AGG CGT ATG TGG CAA AAT-3', 5'-CAT ATA GAA GAT AGA AAA AAA CAG TAC AAT GAC C-3'; and SMN2 full-length, 5'-CAC CAC CTC CCA TAT GTC CAG ATT-3', 5'-GAA TGT GAG CAC CTT CCT TCT TT-3'; Ruggiu et al. 2012) in 1x MESA Green qPCR Master Mix Plus (Nizzardo et al. 2014). The reactions and recording were performed on an Applied Biosystem 7500 Fast Real-Time PCR System in technical duplicates. The specificity of signals was controlled by recording dissociation curves following the amplification. CT values were extracted with the accompanying software and signals below lower detection limits and with unspecific amplification were deemed non-detected. The

relative expression levels were calculated according to the ΔCT method (Pfaffl 2001). For the RNA-seq data, the aligned BAM files were merged per cell type and genotype (combining all time points). Sashimi plots were generated using the Integrative Genomics Viewer software.

RNA-seq data analysis

For principal component analysis and gene expression heatmaps, data was log_2-transformed. Weighted gene correlation network analysis (WGCNA) (Langfelder and Horvath 2008) was performed using control somatic motor neuron samples to investigate developmental gene expression changes, and control and SMA somatic motor neurons for the analysis of SMA induced transcriptional changes. To eliminate expression noise, the gene sets included in the analyses were limited to genes expressed at RPKM ≥ 1 in at least three replicates of any sample type (i.e. consistently expressed in at least one group of cells with common genotype, cell type and time point). Unsigned co-expression networks were constructed for module detection. Module-trait correlations were calculated for categorical, binary traits. Examples of such "traits" could be cell type, genotype, timepoint or a combination of two or more of those categories. Each sample was given a value of 0 or 1 for each trait, depending on which group(s) it belongs to. Figure 3C and Supplemental Fig. S6B show the mean eigengene values (first principal component of the modules) within replicates. Differential expression was calculated using DESeq2 (Love et al. 2014) after first removing all genes with zero expression across all samples and adding a pseudo-count of 1. The pseudo-count was added, as DESeq2 appeared to perform poorly when most replicates had a zero count. Independent filtering (independentFiltering = FALSE) and outlier filtering (cooksCutoff = FALSE) were disabled as their inclusion occasionally resulted in our control gene (*Smn*) lacking significance. Events were considered significant when the adjusted *P*-value was below 0.05. As the biological importance of a given change in expression level is unknown, no fold change cut-off was applied. To further control for cell-type driven differences, we performed DESeq2 analysis between CN3/4 samples and samples of CN7, CN12 and SC, both for SMA and control. In the control condition, this essentially reflects cell type differences. We subsequently isolated genes that were highly significant (P_{adj} <0.01) in the SMA condition between CN3/4 versus CN7, CN3/4 versus CN12 and CN3/4 versus SC, but not nearly significant (P_{adj} >0.5) in the control condition. Gene overlaps were calculated

in R using the VennDiagram package (version 1.6.17). GO term analysis for biological processes, molecular function and cellular compartment was conducted with Panther (http://www.pantherdb.org/) (Mi et al. 2013) (Panther Overrepresentation Test, release 2017-04-13; GO Ontology database release 2017-09-26; Bonferroni correction of *P*-values). To retrieve protein-protein interaction networks, we used the STRING database (https://string-db.org/, version 10.5 (Szklarczyk et al. 2017)) and uploaded selected gene sets using the 'Multiple proteins' query. The minimum required interaction score was set to 0.04 (medium confidence) and the interaction sources neighborhood and gene fusion were deselected. For the identification of genes that belong to certain GO terms (Supplemental Fig. S10-12) complete gene lists of these terms (filtered for *Mus musculus*) were downloaded from AmiGO 2 (http://amigo.geneontology.org/amigo) (The Gene Ontology Consortium 2017; Ashburner et al. 2000).

Neuromuscular junction analysis

For neuromuscular junction (NMJ) immunohistochemistry of extraocular, lumbrical (from the hind-paw) and tongue muscles, tissue was dissected in 0.1 M phosphate buffered saline (PBS, LifeTechnologies) and post-fixed for 30 and 60 min, respectively, in 4% paraformaldehyde (PFA, Sigma-Aldrich). Lumbrical muscles were left intact for whole-mount processing. To increase visible muscle surface, muscles were gently stretched laterally. Tongues were sectioned at 30 µm thickness. For visualization of NMJs, tissue was permeabilized in 4% Triton X-100 in 0.1 M PBS for 1 hour and blocked in 0.1% Triton X-100 in 10% donkey serum in 0.1 M PBS for 1 hour at room temperature. To label the NMJ and incoming axons, tissue was incubated with an anti-neurofilament (165 kDa) antibody (2H3, 1:50 (Dodd et al. 1988)) and an anti-Synaptic vesicle protein 2 antibody (SV2, 1:100 (Buckley and Kelly 1985)) at 4°C overnight. After washing in blocking solution for 1 hour at room temperature, muscles were incubated for 3 hours in Alexa Fluor 488-conjugated secondary antibody (1:500, LifeTechnologies), followed by washing in 0.1 M PBS for 30 minutes. Subsequently, muscle tissues were incubated with α-bungarotoxin (α-BTX) conjugated to tetramethyl-rhodamine isocyanate (TRITC, 1:1000; Life Technologies) for 10 min in order to visualize post-synaptic acetylcholine receptors (Comley et al. 2016). Muscles were whole-mounted on glass slides in Mowiol 4-88 (Sigma-Aldrich) and cover-slipped. Muscle preparations were imaged using a laser scanning confocal microscope (Zeiss

LSM700 and LSM800). All images were acquired as Z-stacks to ensure all incoming axons were visible. Images are shown as maximum intensity projections. NMJs were quantified as mono-innervated when only a single axon was observed, and as poly-innervated when 2 or more axons connected to the endplate. For perforation analysis, gaps in α–BTX staining within the endplate were quantified (Supplemental Fig. S7B). All NMJ quantifications were only performed on 'en-face' endplates to avoid mis-quantification due to the imaging angle. All analyses were performed blind to the genetic status of the material. Statistical analysis was conducted in GraphPad Prism 6. We performed unpaired, two-tailed *t*-tests and *P*-values were corrected for multiple comparisons with the Holm-Sidak method, with the exception of the analysis of extraocular muscles in Supplemental Fig. S7A, where only one time point was investigated. Multiple testing correction for poly-innervation status was performed across time points, whereas for perforation studies, correction was applied across perforation status within each time point. The number of NMJs analyzed are detailed in Supplemental Tables S3-4.

Motor neuron generation from human iPSCs

A human control iPSC line (BJ) was used. This line was generated at the Harvard Stem Cell Institute from commercially available fibroblasts (Warren, 2010).

Stem cells were grown on tissue-culture treated dishes coated with laminin-521 (BioLamina) and cultured in mTeSR media (StemCell Technologies). Stem cells were passaged after a 30-minute pre-incubation with 5 μM Rock-inhibitor (Y-27632; Tocris) and after replating, Rock-inhibitor was maintained in the media until the next day.

To differentiate iPSCs into motor neurons we adapted a protocol from Guo et al (2017), see also Nijssen et al. 2019. The iPSCs were dissociated to single cells with TrypLE Express and resuspended in N2/B27 media (DMEM-F12 supplemented with 1x N2 in a 1:1 ratio with Neurobasal media supplemented with 1x B27, all Thermo Fisher Scientific). During the entire differentiation procedure and subsequent motor neuron culture the media contained 200 μM ascorbic acid (Sigma-Aldrich). For the first 2 days, the differentiation media was supplemented with 5 μM Rock-inhibitor, 40 μM SB-431542, 200 nM LDN-193189 and 3 μM CHIR99021 (all Tocris). As of the third day, the media was instead supplemented with 200 nM retinoic acid (RA, Sigma-Aldrich) and 500 nM smoothened agonist (SAG, Peprotech). On day 10 the EBs were dissociated and plated in 96 well plates at 17,000 cells per well. Upon plating, the

media was changed to B27 only, supplemented with 200 nM retinoic acid for one more day, 10 µM DAPT (Tocris) for four days and 10 ng/ml of both brain-derived neurotrophic factor (BDNF, Peprotech) and glial cell-derived neurotrophic factor (GDNF, Peprotech). After four days (day 14), the media consequently contained only BDNF, GDNF and ascorbic acid and media was changed every other day.
After one more week, at day 21, BDNF and GDNF were removed from the media. From this point onwards, the motor neurons were treated with varying concentrations of GDF15 (Peprotech).

Immunocytochemistry and image analysis of iPSC-derived motor neurons
At various time points, the motor neuron cultures were fixated with 2% paraformaldehyde solution for 20 minutes at room temperature. After washing with PBS, blocking solution was added (10% normal donkey serum and 0.1% Triton X-100 in PBS) for 1 hour at room temperature. Subsequently incubation with primary antibodies was conducted in the same solution for 2 overnights at 4°C. Primary antibodies used were: mouse anti-ISL1/2 (DSHB, 39.4D5) at 1:50 and rabbit anti-Tuj1 (beta-3 tubulin, Biolegend, 802001). After primary antibody incubation, cells were washed with PBS and incubated with Alexa-fluor conjugated secondary antibodies (Thermo Fisher Scientific) for 1 hour at room temperature. Nuclei were counterstained with Hoechst 33342.
The plates were imaged on a LSM800 confocal microscope system (Zeiss) in the Biomedicum Imaging Core (BIC) facility. A total of 4 independent differentiations were performed, with 2-5 replicate wells imaged per condition per experiment. In each well, 12 images were taken in a raster formation, using the nuclear counterstain as reference so as not to bias the imaging. Subsequently, a threshold was applied to images from the nuclear staining and the Islet staining to best label the positive cells, and particles were counted using the ‘analyze particles’ function in Fiji (ImageJ). The number of (Islet-positive) cells was then aggregated per well, and all data is presented as data points per well.

Supplemental Discussion

Our initial analysis of neuronal populations showed that ocular motor neurons had a transcriptional profile that was distinct from other somatic motor neuron groups. This

transcriptional distinction of ocular motor neurons is consistent with previous microarray studies conducted on tissues from control rats (Hedlund et al. 2010), mice (Kaplan et al. 2014) and humans (Brockington et al. 2013). Our PCA also revealed that the global gene expression of ocular motor neurons was more similar to other somatic motor neuron groups and red nucleus neurons than to visceral vagus motor neurons. Red nucleus and ocular neurons display commonalities as these cell types share a developmental origin and are both specified from *Lmx1b*-positive floor plate progenitors, located lateral to the dopamine domain (Deng et al. 2011). Ocular motor neurons share a developmental expression of the transcription factors *Islet1/2*, *Phox2a* and *Phox2b* with other cranial motor neuron groups, including facial and hypoglossal. However, ocular motor neurons uniquely lack the expression of *Hb9* (*Mnx1*) and are not subjected to patterning from Hox genes during development, unlike all other motor neuron groups in the brainstem and spinal cord (reviewed in Cordes 2001). Hox genes are important for establishing motor neuron pool identity as well as connectivity with muscle targets (Dasen et al. 2005) and as such it is not surprising that ocular motor neurons are distinct from other somatic motor neuron groups. Nonetheless, based on the entire gene expression profile, ocular motor neurons were more closely related to somatic motor neurons than to visceral vagus motor neurons.

Additionally, we detected cell-type specific transcripts in several neuronal populations. *Shox2* is a transcription factor involved in pattern formation. We found *Shox2* expression restricted to facial motor neurons in line with the finding that *Shox2* is required for proper development of the facial motor nucleus (Rosin et al. 2015). While *Dcn,* coding for the proteoglycan decorin, was to some degree detectable in facial motor neurons, *Dcn* levels in hypoglossal motor neurons were higher at all time points investigated, revealing this gene as an excellent marker for the hypoglossal nucleus. To the best of our knowledge, there was previously no established marker available that reliably distinguishes hypoglossal from other brainstem motor neurons. Finally, we found *Cxcl13* to be highly restricted to red nucleus neurons, which adds to the existing repertoire of markers for this midbrain nucleus. Thus, we have revealed cell-type specific markers that can be highly valuable tools to selectively modulate cellular functions and identify distinct cell types *in vitro* and *in vivo.*

To our knowledge, no systematic analysis has been performed to evaluate neuron

health in SMA in RN and CN10. An analysis of exceptionally severe (type I) SMA patients (Harding et al, 2015) revealed pathology (chromatolysis) in RN as well as CN10 neurons in the two most severe cases in which the patients died exceptionally early (18 days and 7 weeks of age). However, pathology in these two extremely severe cases expanded to several other areas of the CNS that are classically not considered affected in SMA, making these cases very unusual. The remaining three severe cases did not show such pathology. While there is evidence of some autonomic dysfunction in SMA (reviewed in Shababi et al, 2014), this evidence is based on the investigation of peripheral organs and measurements like blood flow and heart rate. Our LCM-seq data comparing SMA and control does not indicate the activation of stress responses in RN or CN10 in this mouse model, which is considered a model for type I SMA. We do appreciate that we cannot exclude some involvement of these two nuclei in SMA entirely, but it is clear that both RN and CN10 are relatively resilient compared to somatic motor neurons and transcriptional changes are distinct from CN3/4 motor neurons. And thus we conclude that RN and CN10 neurons are relatively unaffected in this SMA model even at later time points. However, a systematic longitudinal analysis of RN and CN10 neuron counts may be warranted to further evaluate their vulnerability status in late stages of SMA.

Consistent with the activation of TP53-signaling, the analysis of SMA and control motor neurons suggests that DNA damage pathways are activated in SMA. This is also in line with previous reports investigating SMA-induced transcriptional changes (Jangi et al. 2017; Murray et al. 2015; Staropoli et al. 2015; Bäumer et al. 2009). Our data support the presence of DNA damage as evidenced by increased expression levels of multiple genes with roles in DNA repair, including *Atr*, involved in the DNA damage response, and commonly regulated in all somatic motor neurons in SMA; the DNA polymerase *Polk* was upregulated in ocular, facial and spinal motor neurons; selectively upregulated transcripts in ocular motor neurons were the DNA repair gene O-6 methylguanine-DNA methyltransferase *Mgmt*, and the tankyrase *Tnks2*, which plays a role in RAD51 recruitment to DNA double strand breaks (Nagy et al. 2016); the RAD51 family member *Rad51d* was upregulated in spinal motor neurons as was *Timeless*, which can mediate DNA damage repair of double strand breaks (Young et al. 2015; Xie et al. 2015). It has been suggested that SMN is important for preventing DNA damage as it associates with RNA pol II (Pellizzoni et al. 2001) and is required

for resolving RNA-DNA hybrids during transcription termination (Zhao et al. 2015). These findings provide a putative mechanism by which DNA damage could occur in SMN deficient cells. While TP53 signaling and especially *Cdkn1a* levels were increased during pre- and early symptomatic disease stages (P2 and P5), expression levels of DNA repair genes were predominantly altered later in disease (P10). Thus, further investigation is warranted to elucidate if DNA damage is a major driver of neuromuscular pathology in SMA.

Direct evidence for DNA damage in SMA motor neurons is as yet limited. Three recent studies failed to detect increased γH2AX (H2AX phosphorylated on serine 139) immunoreactivity, a reliable marker for DNA double strand breaks, in motor neurons in SMA mouse tissues and human SMA cells (Simon et al. 2017; Jangi et al. 2017; Murray et al. 2015). Tisdale et al. (2013) observed reduced levels of H2AX mRNA after *Smn*-knockdown and correspondingly lower induction of H2AX phosphorylation upon DNA damage, presenting a possible explanation for the lack of increased γH2AX in SMA motor neurons in the presence of DNA damage. Furthermore, knockdown of *Fus*, which can directly interact with SMN (Yamazaki et al. 2012), led to diminished γH2AX immunoreactivity in response to a genotoxic reagent, while DNA damage was validated using the comet assay (Wang et al. 2013).

Finally, several genes involved in RNA regulation, including *FUS* and TDP-43, which when mutated cause the motor neuron disease amyotrophic lateral sclerosis (ALS), contribute to the prevention or repair of transcription-associated DNA damage. Depletion of FUS or TDP-43 leads to an increase in DNA damage (Hill et al. 2016) and it appears that loss of SMN could have a similar result.

Supplemental References

Ashburner M, Ball CA, Blake JA, Botstein D, Butler H, Cherry JM, Davis AP, Dolinski K, Dwight SS, Eppig JT, et al. 2000. Gene ontology: Tool for the unification of biology. *Nat Genet* **25**: 25–29.

Brockington A, Ning K, Heath PR, Wood E, Kirby J, Fusi N, Lawrence N, Wharton SB, Ince PG, Shaw PJ. 2013. Unravelling the enigma of selective vulnerability in neurodegeneration: Motor neurons resistant to degeneration in ALS show distinct gene expression characteristics and decreased susceptibility to excitotoxicity. *Acta Neuropathol* **125**: 95–109.

Buckley K, Kelly RB. 1985. Identification of a Transmembrane Glycoprotein Specific for Secretory Vesicles of Neural and Endocrine Cells. *J Cell Biol* **100**: 0–10.

Cordes SP. 2001. Molecular genetics of cranial nerve development in mouse. *Nat Rev Neurosci* **2**: 611–23.

Dasen JS, Tice BC, Brenner-Morton S, Jessell TM. 2005. A Hox regulatory network establishes motor neuron pool identity and target-muscle connectivity. *Cell* **123**: 477–491.

Dodd J, Morton SB, Karagogeos D, Yamamoto M, Jessell TM. 1988. Spatial Regulation of Axonal Glycoprotein Expression on Subsets of Embryonic Spinal Neurons. *Neuron* **I**: 105–116.

Hill SJ, Mordes DA, Cameron LA, Neuberg DS, Landini S, Eggan K, Livingston DM. 2016. Two familial ALS proteins function in prevention/repair of transcription-associated DNA damage. *Proc Natl Acad Sci U S A* **113**: E7701–E7709.

Langfelder P, Horvath S. 2008. WGCNA: An R package for weighted correlation network analysis. *BMC Bioinformatics* **9**.

Love MI, Huber W, Anders S. 2014. Moderated estimation of fold change and dispersion for RNA-seq data with DESeq2. *Genome Biol* **15**: 550.

Nagy Z, Kalousi A, Furst A, Koch M, Fischer B, Soutoglou E. 2016. Tankyrases Promote Homologous Recombination and Check Point Activation in Response to DSBs. *PLoS Genet* **12**: 1–26.

Nizzardo M, Simone C, Salani S, Ruepp MD, Rizzo F, Ruggieri M, Zanetta C, Brajkovic S, Moulton HM, Müehlemann O, et al. 2014. Effect of combined systemic and local morpholino treatment on the spinal muscular atrophy δ7 mouse model phenotype. *Clin Ther* **36**: 340–356.

Mi H, Muruganujan A, Casagrande JT, Thomas PD. 2013. Large-scale gene function analysis with the PANTHER classification system. *Nat Protoc* **8**: 1551–1566.

Pellizzoni L, Charroux B, Rappsilber J, Mann M, Dreyfuss G. 2001. A functional interaction between the survival motor neuron complex and RNA polymerase II. *J Cell Biol* **152**: 75–85.

Pfaffl MW. 2001. A new mathematical model for relative quantification in real-time RT-PCR. *Nucleic Acid Res* **29**: 16–21.

Picelli S, Björklund AK, Reinius B, Sagasser S, Winberg G, Sandberg R. 2014a. Tn5 transposase and tagmentation procedures for massively scaled sequencing projects. *Genome Res* **24**: 2033–2040.

Rosin JM, Kurrasch DM, Cobb J. 2015. Shox2 is required for the proper development of the facial motor nucleus and the establishment of the facial nerves. *BMC Neurosci* **16**: 39.

Szklarczyk D, Morris JH, Cook H, Kuhn M, Wyder S, Simonovic M, Santos A, Doncheva NT, Roth A, Bork P, et al. 2017. The STRING database in 2017: Quality-controlled protein-protein association networks, made broadly accessible. *Nucleic Acids Res* **45**: D362–D368.

Shababi M, Lorson CI, Rudnik-Schöneborn SS. 2014. Spinal muscular atrophy: a motor neuron disorder or a multi-organ disease. J Anat 224(1):15-28.

The Gene Ontology Consortium. 2017. Expansion of the Gene Ontology knowledgebase and resources. *Nucleic Acids Res* **45**: D331–D338.

Tisdale S, Lotti F, Saieva L, VanMeerbeke JP, Crawford TO, Sumner CJ, Mentis GZ, Pellizzoni L. 2013. SMN is essential for the biogenesis of U7 Small nuclear ribonucleoprotein and 3'-end formation of Histone mRNAs. *Cell Rep* **5**: 1187–1195.

Wang WY, Pan L, Su SC, Quinn EJ, Sasaki M, Jimenez JC, MacKenzie IRA, Huang EJ, Tsai LH. 2013. Interaction of FUS and HDAC1 regulates DNA damage response and repair in neurons. Nat Neurosci 16: 1383–1391.

Warren L, Manos PD, Ahfeldt T, Loh YH, Li H, Lau F, Ebina W, Mandal PK, Smith ZD, Meissner A et al. 2010. Highly efficient reprogramming to pluripotency and directed differentiation of human cells with synthetic modified mRNA. Cell Stem Cell 7: 618–630

Xie S, Mortusewicz O, Ma HT, Herr P, Poon RRY, Helleday T, Qian C. 2015. Timeless Interacts with PARP-1 to Promote Homologous Recombination Repair. *Mol Cell* **60**: 163–176.

Yamazaki T, Chen S, Yu Y, Yan B, Haertlein TC, Carrasco MA, Tapia JC, Zhai B, Das R, Lalancette-Hebert M, et al. 2012. FUS-SMN Protein Interactions Link the Motor Neuron Diseases ALS and SMA. *Cell Rep* **2**: 799–806.

Young LM, Marzio A, Perez-Duran P, Reid DA, Meredith DN, Roberti D, Star A, Rothenberg E, Ueberheide B, Pagano M. 2015. TIMELESS Forms a Complex with PARP1 Distinct from Its Complex with TIPIN and Plays a Role in the DNA Damage Response. *Cell Rep* **13**: 451–459.

Supplemental Table S1. Number of samples analyzed per cell type, time point and genotype.

	Number of samples analyzed (168 total)					
	P2		P5		P10	
Cell population	**control**	**SMA**	**control**	**SMA**	**control**	**SMA**
**RN**	4	4	3	5	4	4
**CN3/4**	4	6	6	6	6	6
**CN7**	5	7	4	3	4	4
**CN10**	3	6	4	3	4	5
**CN12**	4	6	4	4	4	5
**SC**	4	8	5	5	4	5

P= postnatal day; SMA = spinal muscular atrophy, RN = red nucleus, CN3/4 = oculomotor and trochlear (ocular) nuclei, CN7 = facial nucleus, CN10 = dorsal motor nucleus of vagus, CN12 = hypoglossal nucleus, SC = lumbar spinal cord.

Supplemental Table S3. Number of endplates [and animals] analyzed for polyinnervation in muscles of P5 and P10 SMA mice and age-matched litter mate controls.

	EOM		Tongue		Lumbricals	
	**control**	**SMA**	**control**	**SMA**	**control**	**SMA**
**P5**	-	-	205 [5]	215 [5]	191[5]	132 [5]
**P10**	128 [4]	73 [3]	260 [5]	228 [5]	287 [5]	215 [5]

P= postnatal day; SMA = spinal muscular atrophy, EOM = extraocular muscles.

Supplemental Table S4. Number of endplates [and animals] analyzed for perforations in P5, P10 and P14 SMA mice and age-matched litter mate controls.

	EOM		Tongue		Lumbricals	
	**control**	**SMA**	**control**	**SMA**	**control**	**SMA**
**P5**	260 [4]	196 [3]	362 [4]	408 [5]	429 [5]	291 [4]
**P10**	245 [4]	235 [4]	309 [4]	495 [5]	557 [5]	240 [3]
**P14**	158 [3]	360 [4]	209 [3]	310 [4]	319 [5]	343 [4]

P= postnatal day; SMA = spinal muscular atrophy, EOM = extraocular muscles.

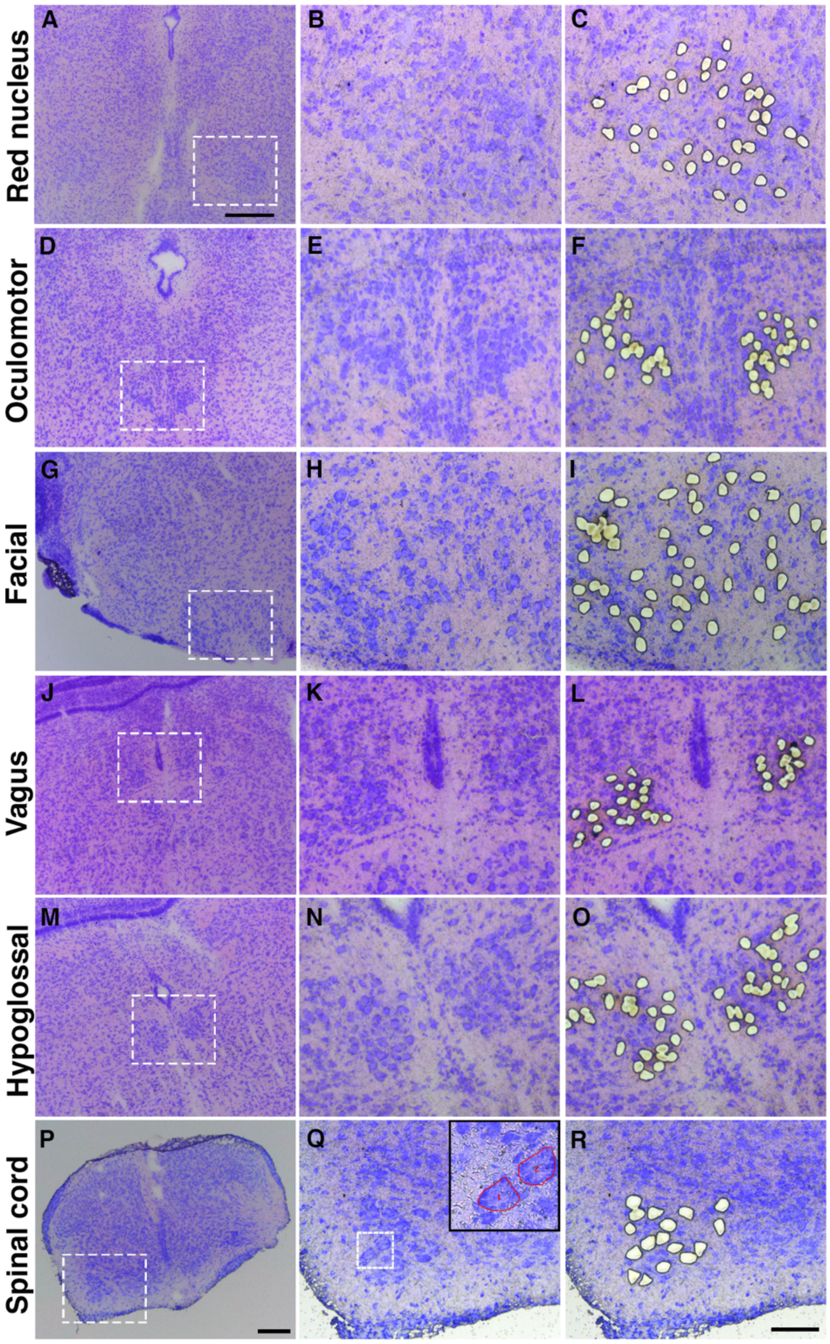

Supplemental Figure S1. Isolation of neuronal populations with differential vulnerability to degeneration in SMA using LCM. Motor neuron populations and red nucleus neurons were identified by their distinct locations in brain and spinal cord and their large size after visualization with a quick histological staining *(A, D, G, J, M, P)*. Images are shown in higher magnification in the middle panel, *(B, E, H, K, N, Q)*, and after LCM in the right panel *(C, F, I, L, O, R)*. In order to minimize contamination with surrounding cells, we drew cutting paths closely around the cells as exemplified in the inset in *Q*. We acquired the example images shown here during several LCM sessions from three different P2 mice. Scale bar in *A* = 400 µm, *P* = 200 µm (applies to *D, G, J, M* and *P*), and *R* = 100 µm (applies to all images in the middle and right panels).

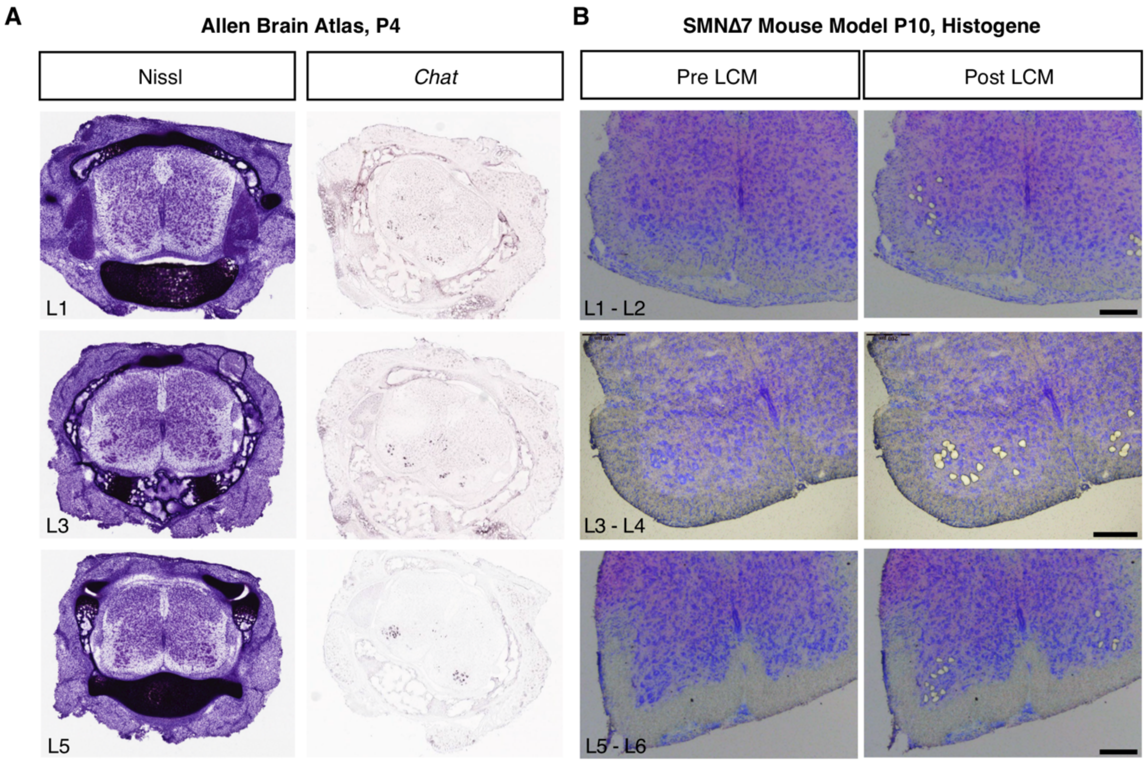

Supplemental Figure S2. Motor neuron isolation from lumbar spinal cord. *(A)* Images from the Allen Brain Atlas showing Nissl stained and *Chat in situ* sections at different levels of the lumbar spinal cord. (© 2004 Allen Institute for Brain Science. Allen Mouse Brain Atlas. Available at: http://mouse.brain-map.org/). *(B)* Examples of images acquired during different LCM sessions of P10 spinal cords pre and post dissection. Motor neurons were collected from all levels of the lumbar spinal cord, and cells from the medial and lateral motor columns were included when possible. Scale bar in *B* = 200 μm.

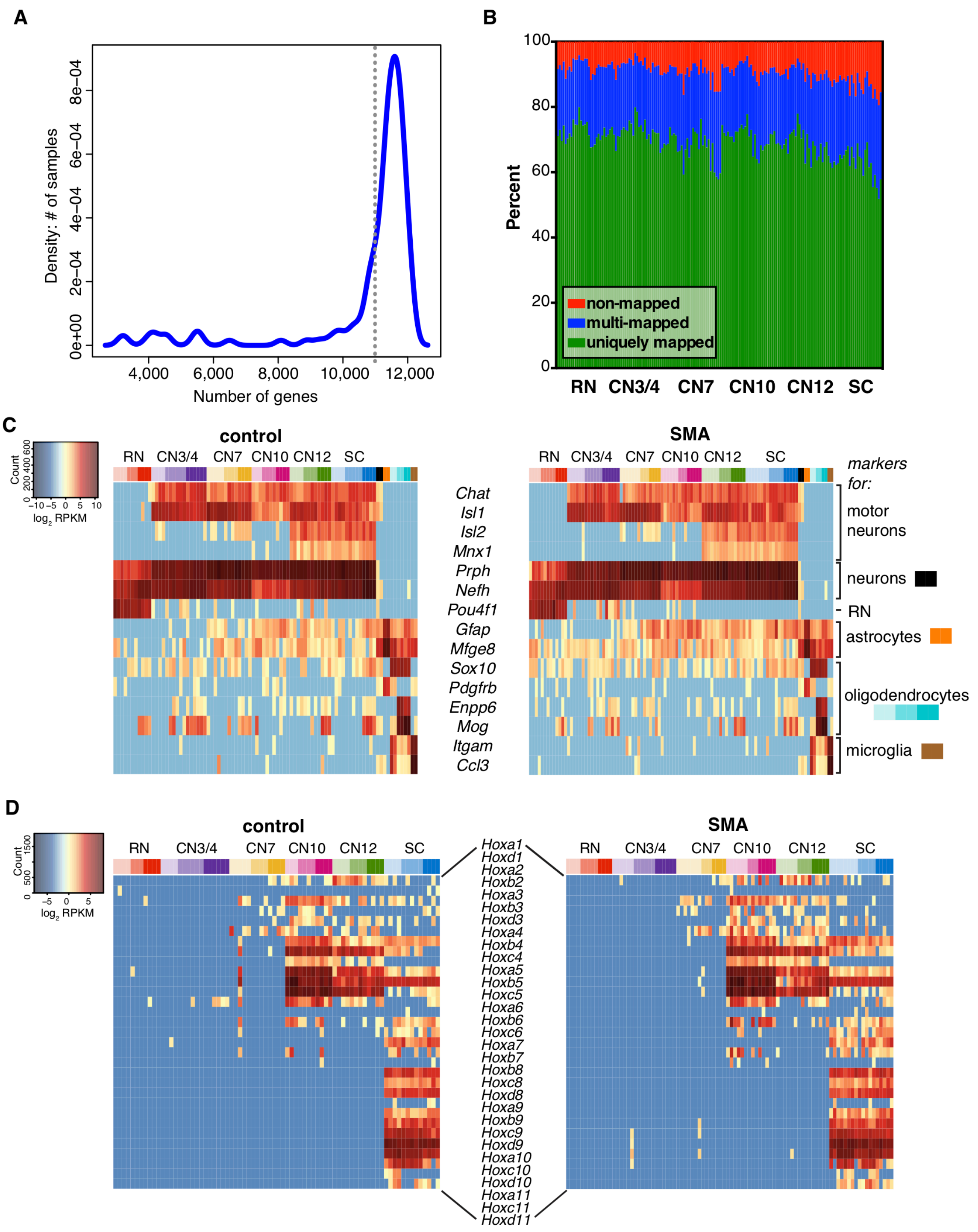

Supplemental Figure S3. Quality control of LCM-seq gene expression data. *(A)* Gene detection level (≥1 RPKM). Samples with less than 11,000 expressed genes were excluded from further analysis. *(B)* Mapping data to the mouse genome (mm10) with an added human *SMN2* sequence. Only uniquely mapped reads (green) were used in the analysis. *(C)* LCM-seq samples were compared to a previously published RNA-seq data set of neurons (black), astrocytes (orange), oligodendrocyte precursor cells (OPCs, light turquoise), newly formed oligodendrocytes (NFO, medium turquoise) and myelinating oligodendrocytes (MO, turquoise) to evaluate the purity of our neuronal samples (GEO accession number GSE52564). *(D)* Hox gene expression profiles to evaluate the positional identity of our neuronal samples. Gene labels are shifted to increase legibility.

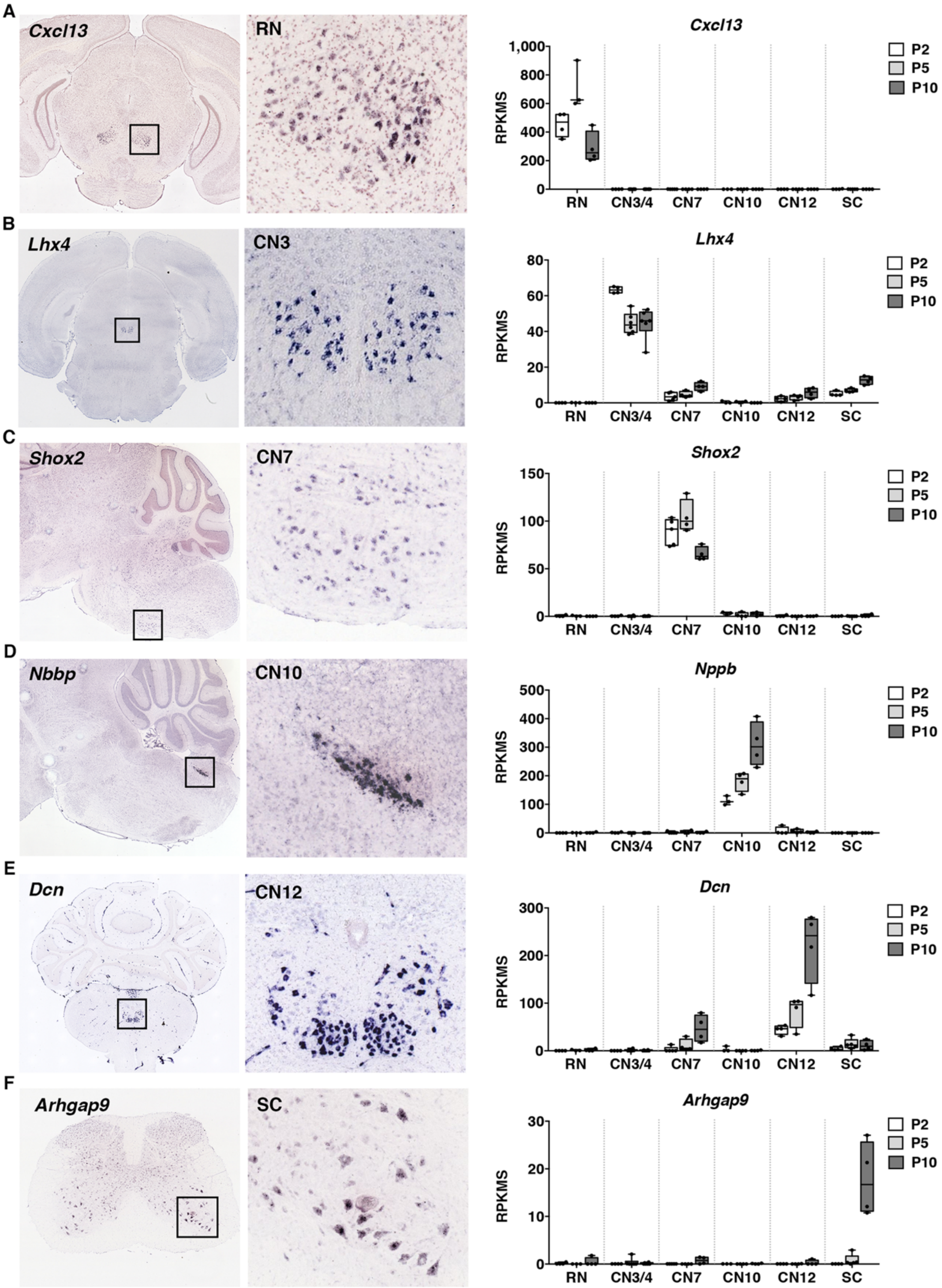

Supplemental Figure S4. Confirmation of cell-type specific expression of selected genes in the adult nervous system. We found *(A) Cxcl13* to be specifically expressed in RN, *(B) Lhx4* in CN3/4, *(C) Shox2* in CN7, *(D) Nbbp* in CN10, *(E) Dcn* in CN12, and *(F) Arhgap9* in SC (© 2004 Allen Institute for Brain Science. Allen Mouse Brain Atlas. Available at: http://mouse.brain-map.org/). Images are not to scale and were adjusted to give a complete overview of the whole brain section (left panel) or the particular nervous system nucleus (middle panel). Expression levels in control samples at P2, P5 and P10 (right panel). RPKMS = reads per kilobase million; boxplots with individual values (dots) and whiskers representing minimum and maximum.

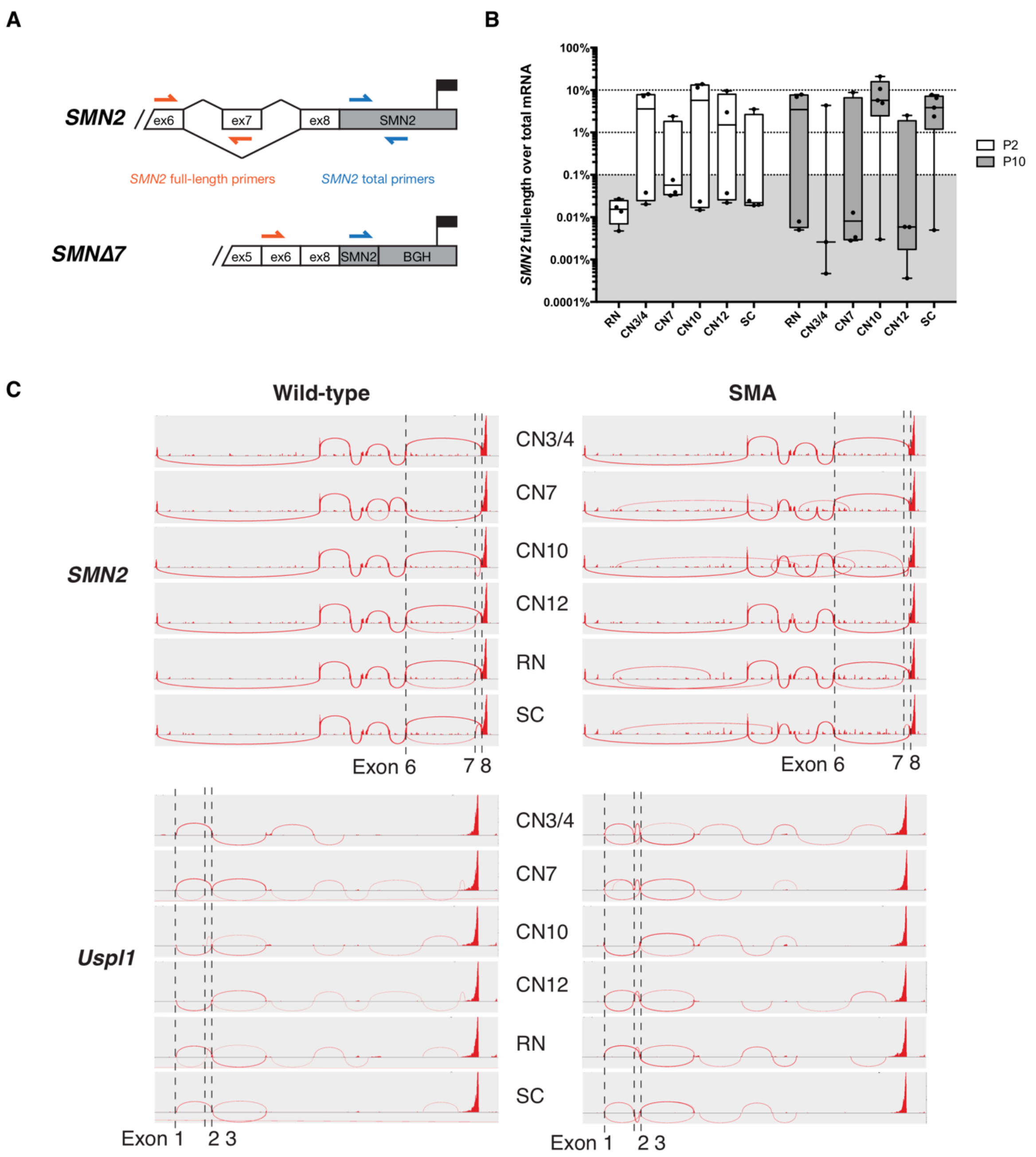

Supplemental Figure S5. SMN2 alternative splicing analysis across neuronal populations. *(A)* A schematic of the primers used to detect full-length (including exon 7) and total *SMN2* mRNA specifically. The assays do not detect the *SMNΔ7* transgene that lacks exon 7 and the *SMN2* 3' UTR. Exons are shown as boxes that are white for the open-reading frame and grey for the 3' UTRs originating from the human *SMN2* and bovine growth hormone (BGH). The polyadenylation signal is indicated by a flag symbol and the primers as arrows. *(B)* Exon 7 inclusion was quantitated by qPCR with the *SMN2* mRNA specific assays and expressed as a ratio of full-length to total *SMN2* mRNA. The proportion of full-length *SMN2* does not exceed on average 10%, while also not being proportionally higher in the resistant neuronal pools compared to the vulnerable ones. The grey region indicates the lower detection limit (background amplification level in water control) with samples lacking detectable exon 7 inclusion. *(C)* Sashimi plots showing junction-spanning reads for *SMN2* and *Uspl1*, a known alternative splicing target for SMN. No clear differences are observed in the rate of *SMN2* exon 7 inclusion between the different neuronal pools. Loss of functional SMN protein does result in a widespread inclusion of exon 2 of *Uspl1*, a control to detect splicing event changes between the wild-type and SMA models.

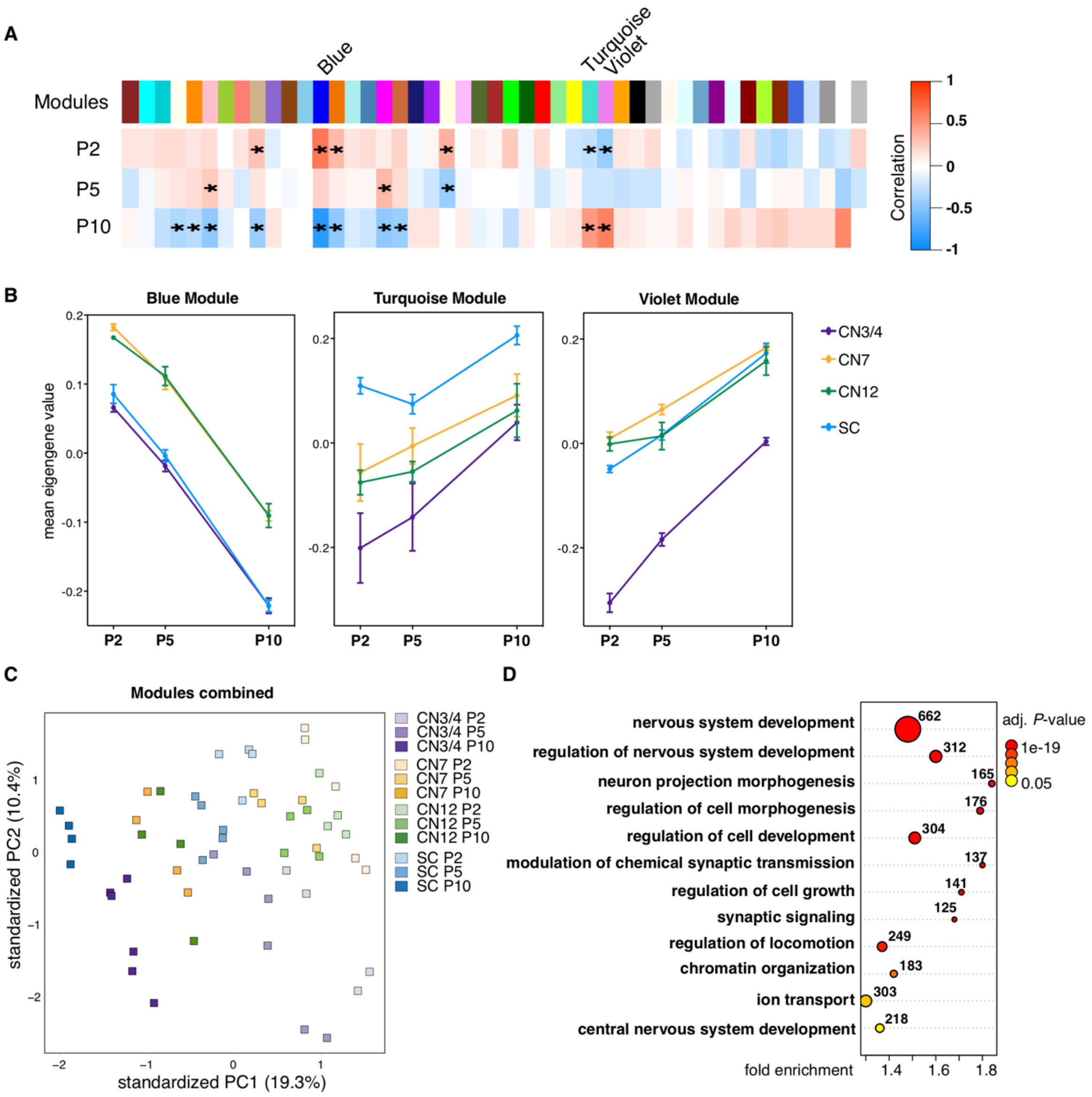

Supplemental Figure S6. Identification of gene sets important for postnatal motor neuron development. *(A)* Module- trait- correlation heatmap for all modules identified by WGCNA using control somatic motor neuron samples. Asterisks indicate statistical significance, $P_{adj.}$<0.05. Modules with highest correlations that are changing over time were chosen. *(B)* Mean eigengene values (first principal components of the three modules) within replicates. *(C)* PCA of the three modules combined (5,843 genes). *(D)* We performed Gene Ontology (GO) term analysis for biological processes and selected terms related to development are depicted here. We used only genes in these terms for analysis shown in Fig. 2. Numbers indicate the number of genes in a given term, color scale = adjusted *P*-value.

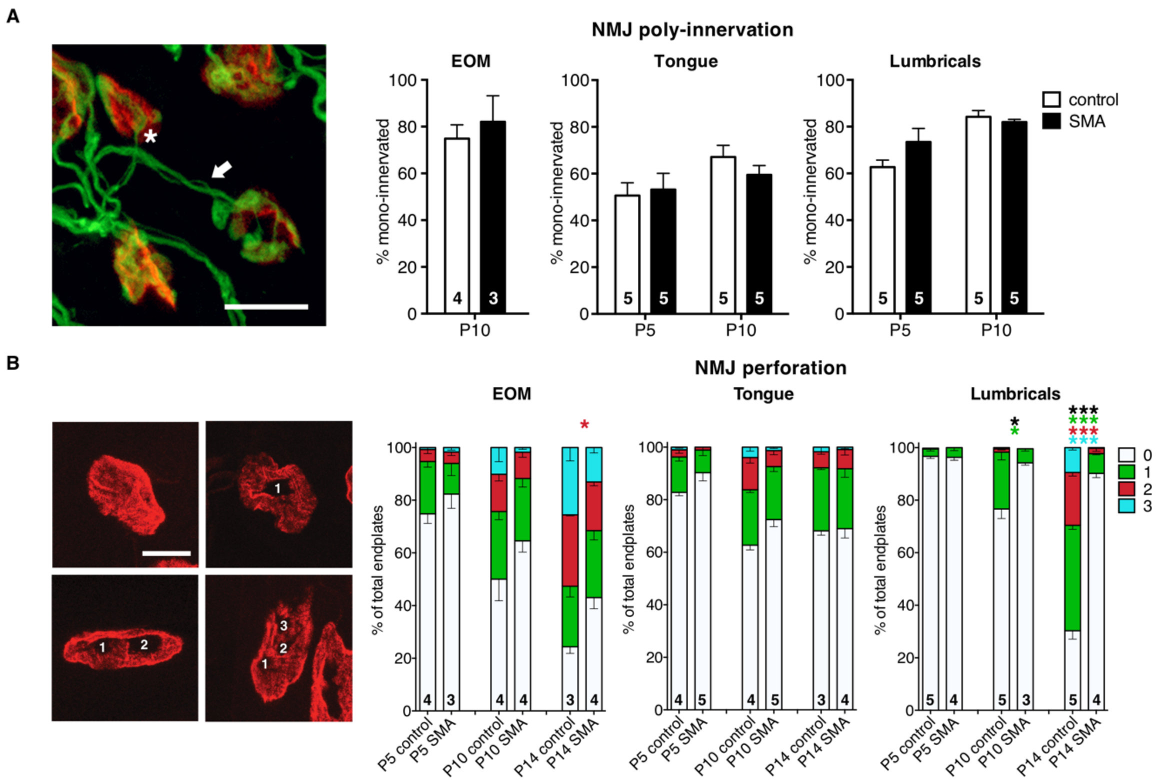

Supplemental Figure S7. Evaluation of the developmental state of SMA peripheral synapses. *(A)* Quantification of neuromuscular junction (NMJ) poly-innervation of extraocular muscles (EOM), tongue and lumbrical muscles. Micrograph shows examples of a mono-innervated (asterisk) and a poly-innervated (arrow) endplate. Red = alpha-bungarotoxin, post-synaptic nicotinic acetylcholine receptors; green = pre-synaptic, Neurofilament 165 kDa (2H3) and Synaptic vesicle protein 2 (SV2). *(B)* Quantification of NMJ perforation. Micrographs show examples of NMJs with 0, 1, 2 and 3 perforations. Red = alpha-bungarotoxin, post-synaptic nicotinic acetylcholine receptors. Numbers on bars in *A* and *B* represent the numbers of animals used for quantification. Multiple two-tailed *t*-tests per group (except for EOM in *A* where only one time point was investigated), * $P_{adj.}$<0.05, **** $P_{adj.}$<0.0001. Scale bar in *A* = 10 µm and in *B* = 10 µm (applicable to all micrographs in *B*).

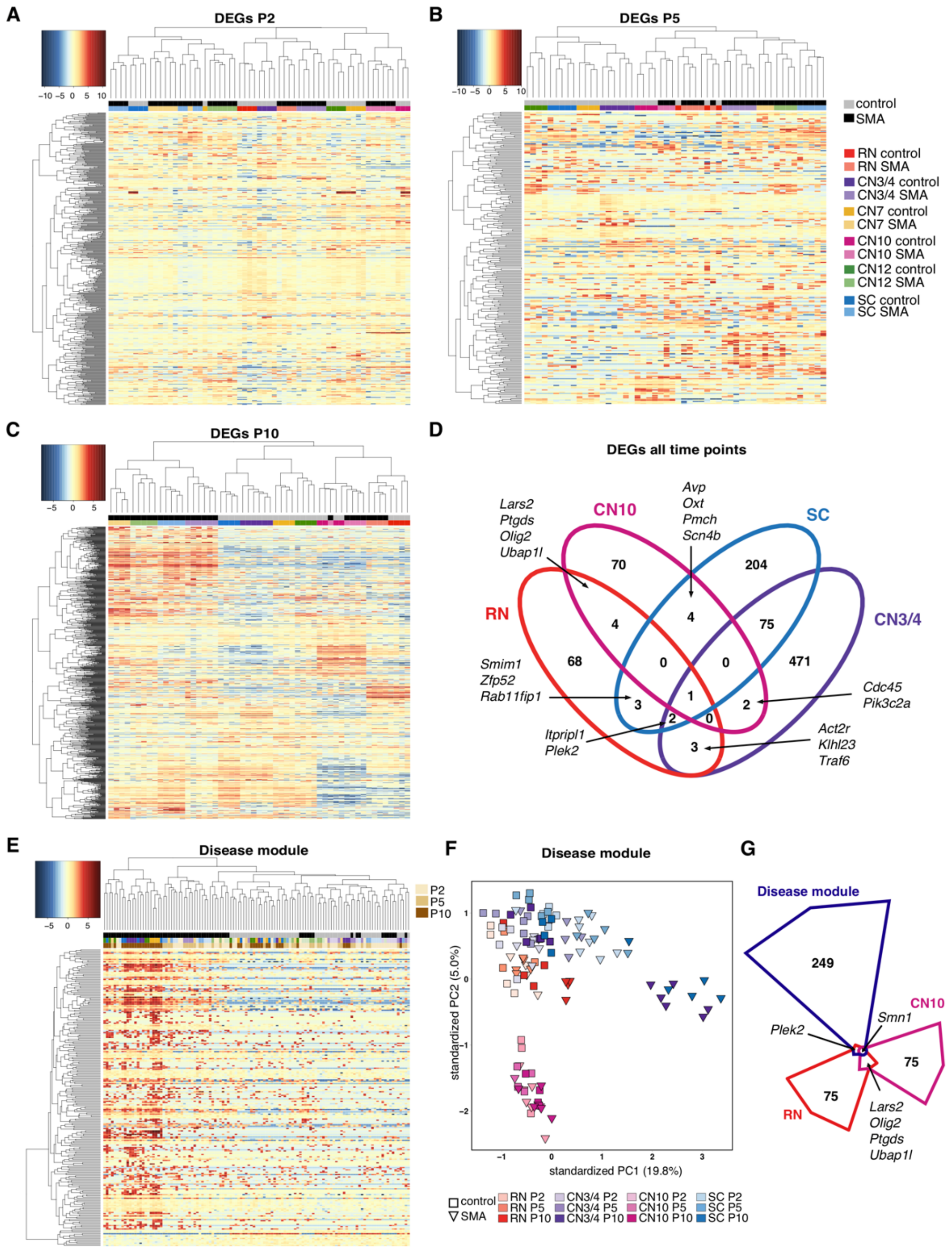

Supplemental Figure S8. Comparison of gene expression changes in all neuron types investigated. Heatmaps of all DEGs between control and SMA from pairwise comparisons per cell type at *(A)* P2, *(B)* P5 and *(C)* P10 (no fold change cut-off, $P_{adj.}$<0.05). *(D)* Venn diagram depicting the overlap in gene expression changes between unaffected controls, resistant CN3/4 and vulnerable SC motor neurons (all time points combined). *(E)* Heatmap of the disease module genes including all somatic motor neuron samples (for genotype and sample color-coding see legend in *B*). *(F)* PCA of RN, CN3/4, CN10 and SC with disease module genes. *(G)* Venn diagram depicting the overlap of RN and CN10 DEGs and the disease module. Expression values for heatmaps were $\log_2$-transformed and mean centered; samples and genes were clustered using Spearman correlation. Legend in *B* applicable to *A, B, C* and *E*.

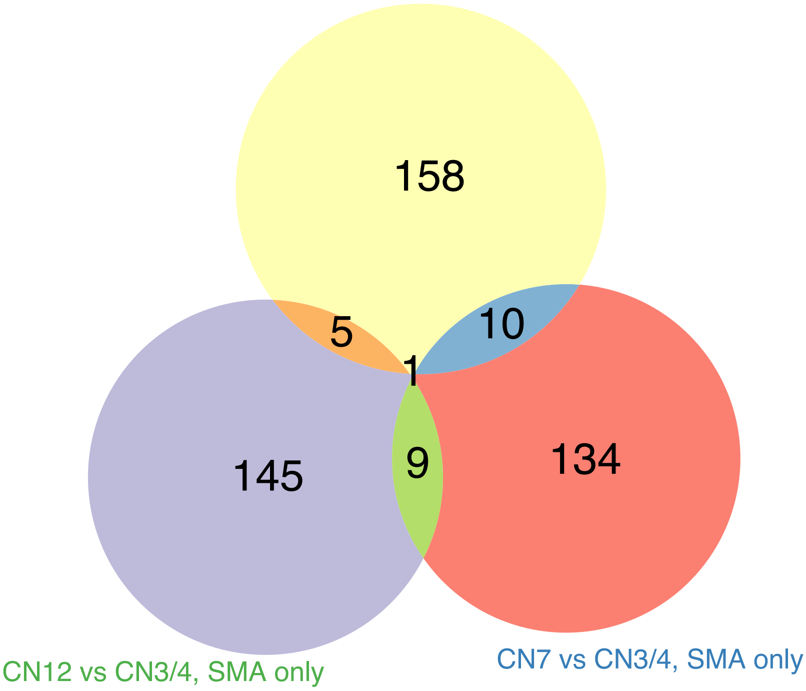

Supplemental Figure S9. SMA-induced gene expression changes between somatic motor neurons. The numbers represent differentially expressed genes in SMA (P_{adj} <0.01) between CN3/4 and either SC, CN7 or CN12, that are not significantly different in the control mice in the same comparisons (P_{adj} >0.05). This removes cell-type driven differences and highlights disease-induced changes between differentially vulnerable neuronal subgroups and ocular motor neurons (CN3/4).

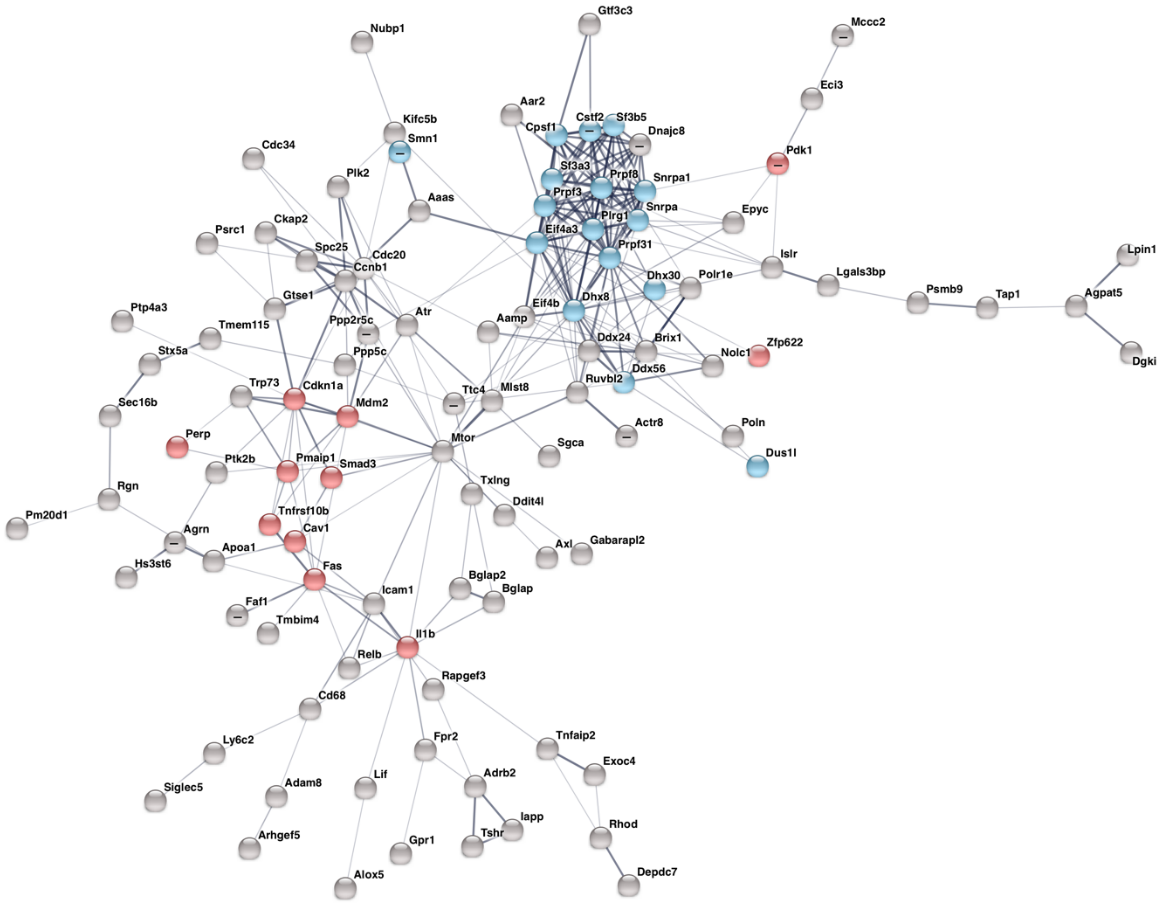

Supplemental Figure S10. Protein-protein interaction network of disease module genes. STRING analysis for protein-protein interactions including all 251 genes in the disease module. Nodes are colored based on selected GO terms: (red) signal transduction by p53 class mediator and apoptotic signaling pathway; (blue) RNA processing. (-) genes with negative module membership, (empty nodes) genes with positive module membership. Network edges represent the confidence of the predicted associations between nodes (edge thickness = strength of data support). Disconnected nodes that do not belong to any of these GO terms are not shown.

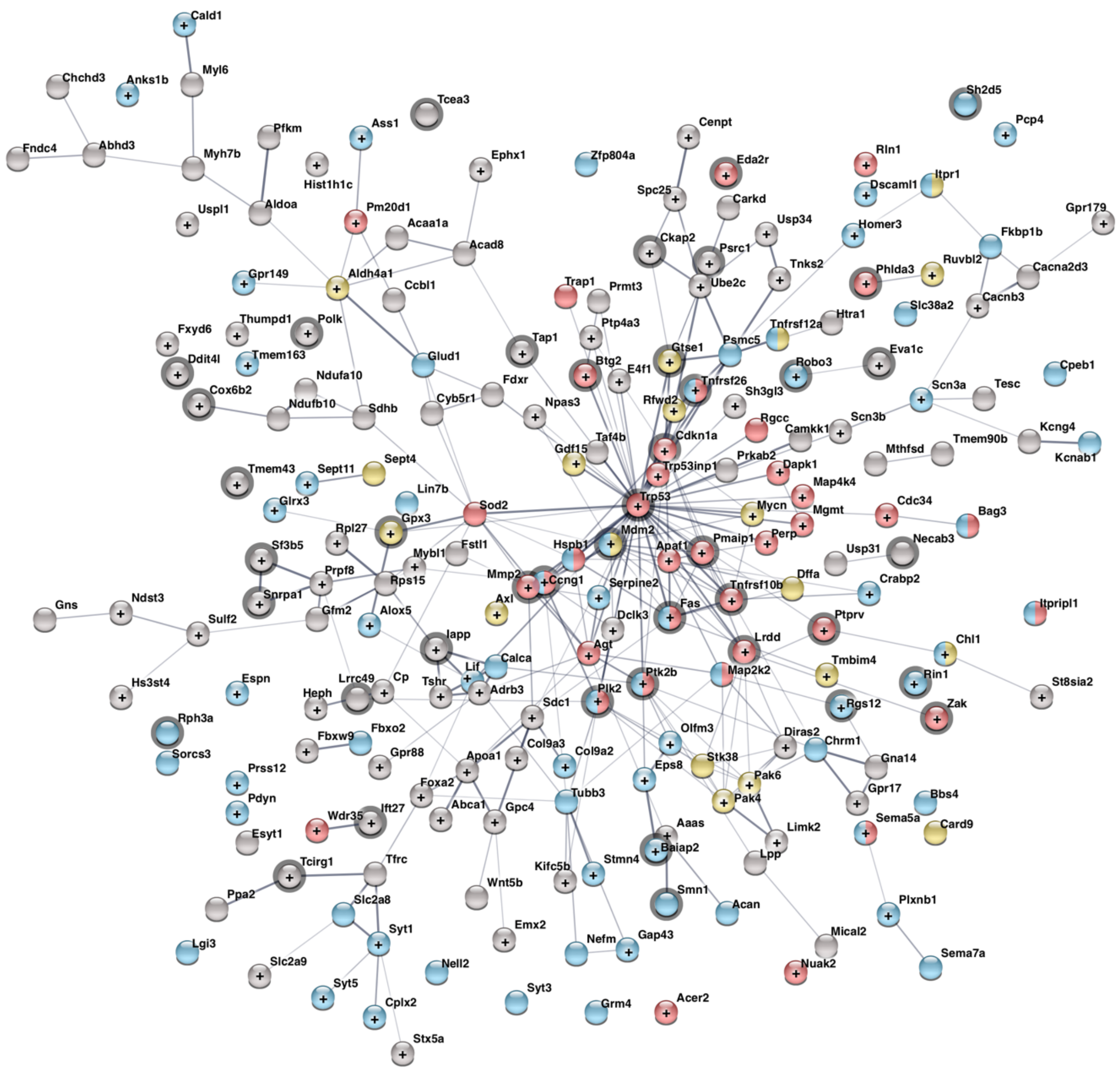

Supplemental Figure S11. Protein-protein interaction network of DEGs in ocular motor neurons at P10. STRING analysis for protein-protein interactions including all 338 DEGs between control and SMA in ocular motor neurons at P10. Nodes are color-coded based on selected GO terms shown in Fig 4C: (red) signal transduction by p53 class mediator and regulation of cell death; (blue) positive regulation of neuron projection development, neuron projection and synapse; (yellow) downregulated apoptotic and upregulated survival genes. (+) upregulated genes in SMA, (empty nodes) downregulated in SMA. (Grey outlines) genes that are also differentially expressed in SC neurons. Network edges represent the confidence of the predicted associations between nodes (edge thickness = strength of data support). Disconnected nodes that do not belong to any of these GO terms are not shown.

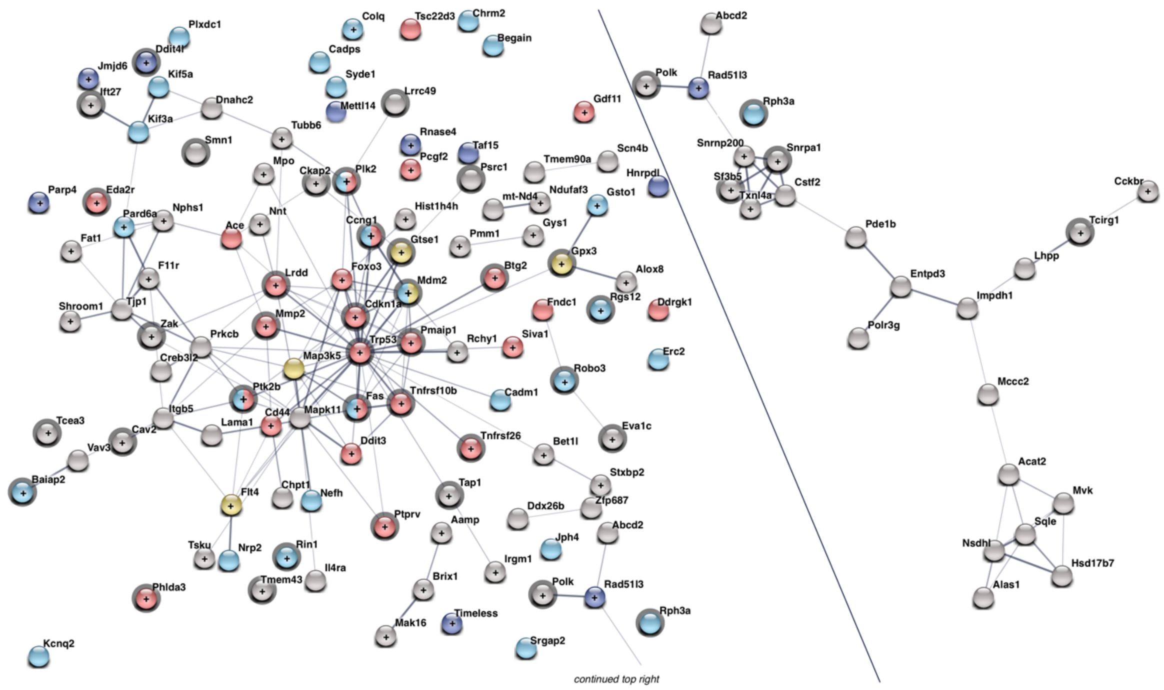

Supplemental Figure S12. Protein-protein interaction networks of DEGs in spinal motor neurons at P10. STRING analysis for protein-protein interactions including all 246 DEGs between control and SMA in spinal motor neurons at P10. Nodes are color-coded based on selected GO terms shown in Fig 4C including CN3/4- specific terms: (red) signal transduction by p53 class mediator and regulation of cell death; (blue) positive regulation of neuron projection development, neuron projection and synapse; (yellow) downregulated apoptotic and upregulated survival genes. (+) upregulated genes in SMA, (empty nodes) downregulated in SMA. (Grey outlines) genes that are also differentially expressed in CN3/4 neurons. Network edges represent the confidence of the predicted associations between nodes (edge thickness = strength of data support). Disconnected nodes that do not belong to any of these GO terms are not shown except for selected genes (dark blue).
